# Author Correction: Massively parallel in vivo CRISPR screening identifies RNF20/40 as epigenetic regulators of cardiomyocyte maturation

**DOI:** 10.1038/s41467-021-25373-1

**Published:** 2021-08-19

**Authors:** Nathan J. VanDusen, Julianna Y. Lee, Weiliang Gu, Catalina E. Butler, Isha Sethi, Yanjiang Zheng, Justin S. King, Pingzhu Zhou, Shengbao Suo, Yuxuan Guo, Qing Ma, Guo-Cheng Yuan, William T. Pu

**Affiliations:** 1 Department of Cardiology, Boston Children’s Hospital, Boston, MA USA; 2 Department of Pharmacology, School of Pharmacy, Shanghai University of Traditional Chinese Medicine, Shanghai, China; 3 Department of Biochemistry, West China School of Basic Medical Sciences & Forensic Medicine, Sichuan University, Chengdu, Sichuan China; 4 Department of Pediatric Oncology, Dana-Farber Cancer Institute, Boston, MA USA; 5 Department of Genetics and Genomic Sciences and Institute for Personalized Medicine, Icahn School of Medicine at Mount Sinai, New York, NY USA; 6 Harvard Stem Cell Institute, Cambridge, MA USA

**Keywords:** Genetic techniques, Differentiation, Histone post-translational modifications, Heart stem cells, Cardiovascular biology

Correction to: *Nature Communications* 10.1038/s41467-021-24743-z, published online 21 July 2021.

The original version of this Article contained an error in the spelling of the author William T. Pu, which was incorrectly given as William William Pu. This has now been corrected in both the PDF and HTML versions of the Article.

