# Supplementary material for: Massively parallel in vivo CRISPR screening identifies RNF20/40 as epigenetic regulators of cardiomyocyte maturation
Source: Nat Commun. 2021 Jul 21;12:4442. doi: 10.1038/s41467-021-24743-z (PMC8295283; doi:10.1038/s41467-021-24743-z)
Supplement: Supplementary file 1 — Supplementary Information [file 41467_2021_24743_MOESM1_ESM.pdf]

Massively parallel in vivo CRISPR screening identifies RNF20/40 as epigenetic regulators of cardiomyocyte maturation

Nathan J. VanDusen, et al.

SUPPLEMENTARY INFORMATION

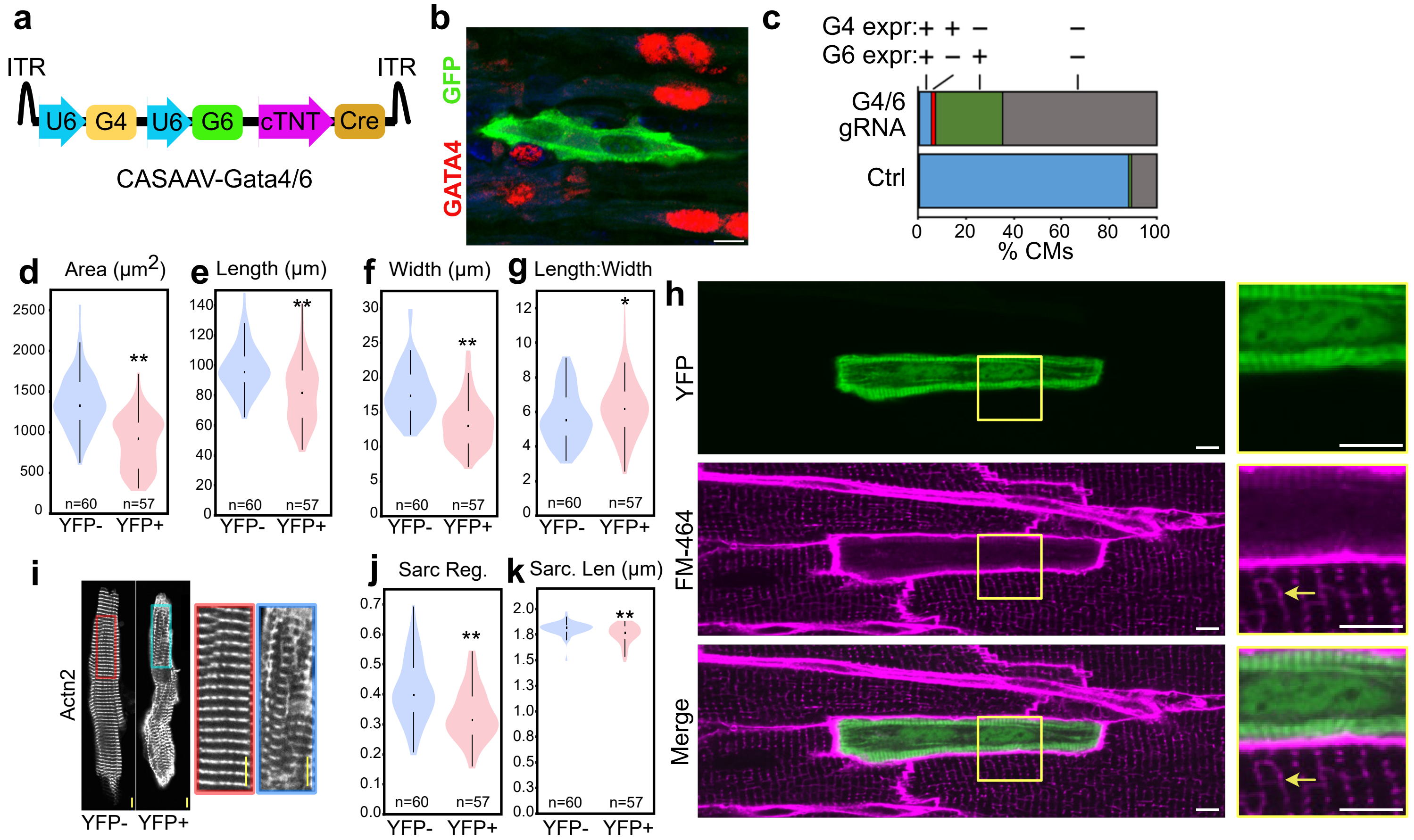

Suppl. Fig. 1. GATA4 and GATA6 are required for normal CM maturation. a. CASAAV-Gata4/6 vector. AAV genome expresses Cre from the CM selective cardiac troponin T (cTNT) promoter and gRNAs that target *Gata4* and *Gata6*. We administered CASAAV-Gata4/6 at a low dose, to achieve mosaic gene inactivation, to postnatal day 1 (P1) mouse pups carrying Cre-activated Cas9-2A-GFP and $Myh7^{YFP}$ ($R26^{Cas9\text{-}GFP;}$ $Myh7^{YFP}$). **b.** GATA4 and GATA6 knockout efficiency. We fixed hearts at P10 and performed GATA4 and GATA6 immunostaining (n=3 hearts). Representative section stained for GATA4 is shown. GFP marked transduced CMs. Scale bar = 10 µm. **c.** Quantification of GATA4 and GATA6 depletion. Greater than 90% and 60% of the transduced CMs lost GATA4 and GATA6 immunoreactivity, respectively. **d-g.** Quantitation of size and dimensions in isolated P28 CMs, when maturation is normally largely complete. Transduced CMs expressing the immaturity marker YFP were compared to YFP- CMs from the same heart. YFP+ CMs were much smaller in projected area (d; P<0.0001), due to decreases in both length and width (e-g; length and width P<0.0001; length-to-width P=0.0409). **h**. T-tubulation was assessed by optical sectioning of intact hearts perfused with the plasma membrane binding dye FM4-64. Severe T-tubule defects were observed in YFP+ cells as the lack of internal FM4-64 stained striations, labeled by arrows in control cells (n=3 mice). Scale bars = 5 µm. **i-k.** Sarcomere organization (YFP- n=60 CMs; YFP+ n=57 CMs). α-actinin (ACTN2) immunostaining of dissociated CMs showed decreased sarcomere regularity within YFP+ CMs, as quantified by AutoTT software. Scale bars = 5 µm. (**i,j;** P<0.0001). Sarcomere spacing was also decreased in YFP+ cells (**k;** P=0.0005). Source data are provided as a Source Data file. Violin plots: shape indicates data distribution; point, median; whiskers, starts at quartile and extends 1.5 times the interquartile distance. Two-tailed t-test: *P<0.05, **P<0.001.

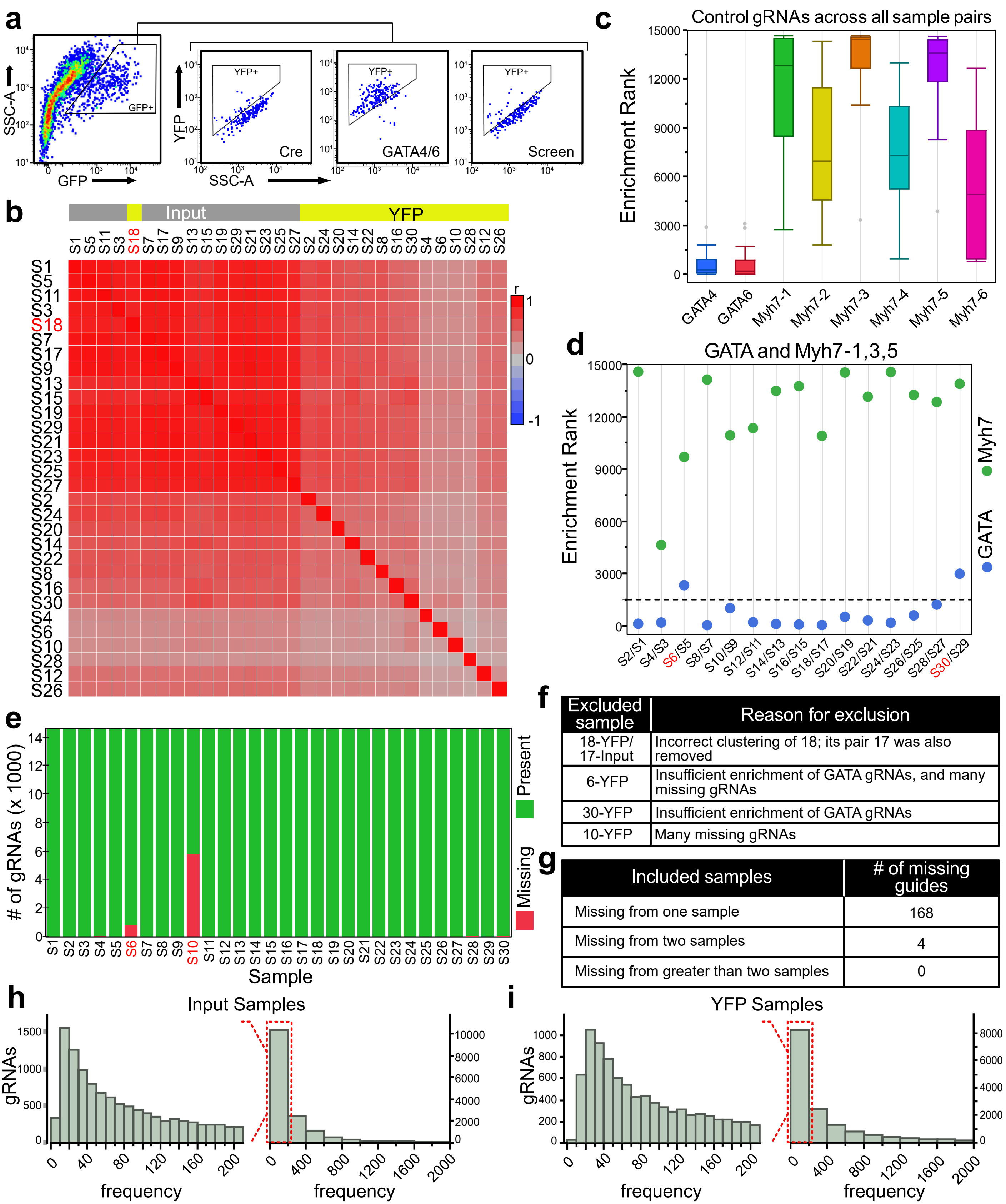

Suppl. Fig. 2. Quality control of forward genetic screen biological replicates. a. Representative flow cytometry plots. First GFP+ CMs were gated (left panel), and then sorted for YFP expression (right three panels). Cre, negative control. GATA4/6, CASAAV-Gata4_Gata6 positive control. Screen, representative example of one of the samples in the screen. **b.** gRNA counts were extracted and the enrichment of gRNAs in YFP samples was calculated for each sample pair. S18 is a YFP sample that mis-clustered with the input samples. **c,d.** Enrichment rank (low=most enriched) of positive (Gata4 and Gata6) and negative (Myh7-1 to Myh7-6) control gRNAs across all sample pairs (c; n=15 for each gRNA) and individual sample pairs (d). Box polot center line indicates the median, while boxes show 25th and 75th percentiles. Whiskers denote the maximum observation within the 75th percentile + 1.5 times the interquartile range, or the minimum observation within the 25th percentile - 1.5 times the interquartile range. **e.** gRNA coverage across sampes. "Missing" guides were guides with zero reads. S6 and S10 had many missing gRNAs. **f.** Excluded samples and reasons for their exclusion. **g.** Consistency of gRNA detection accross all included samples. "Missing" guides were guides with zero reads. **h,i.** Frequency of gRNAs in input (h) and YFP (i) samples. Left panel is a magnification of the first bar in the right panel. Frequency represents median normalized read counts. Source data are provided as a Source Data file.

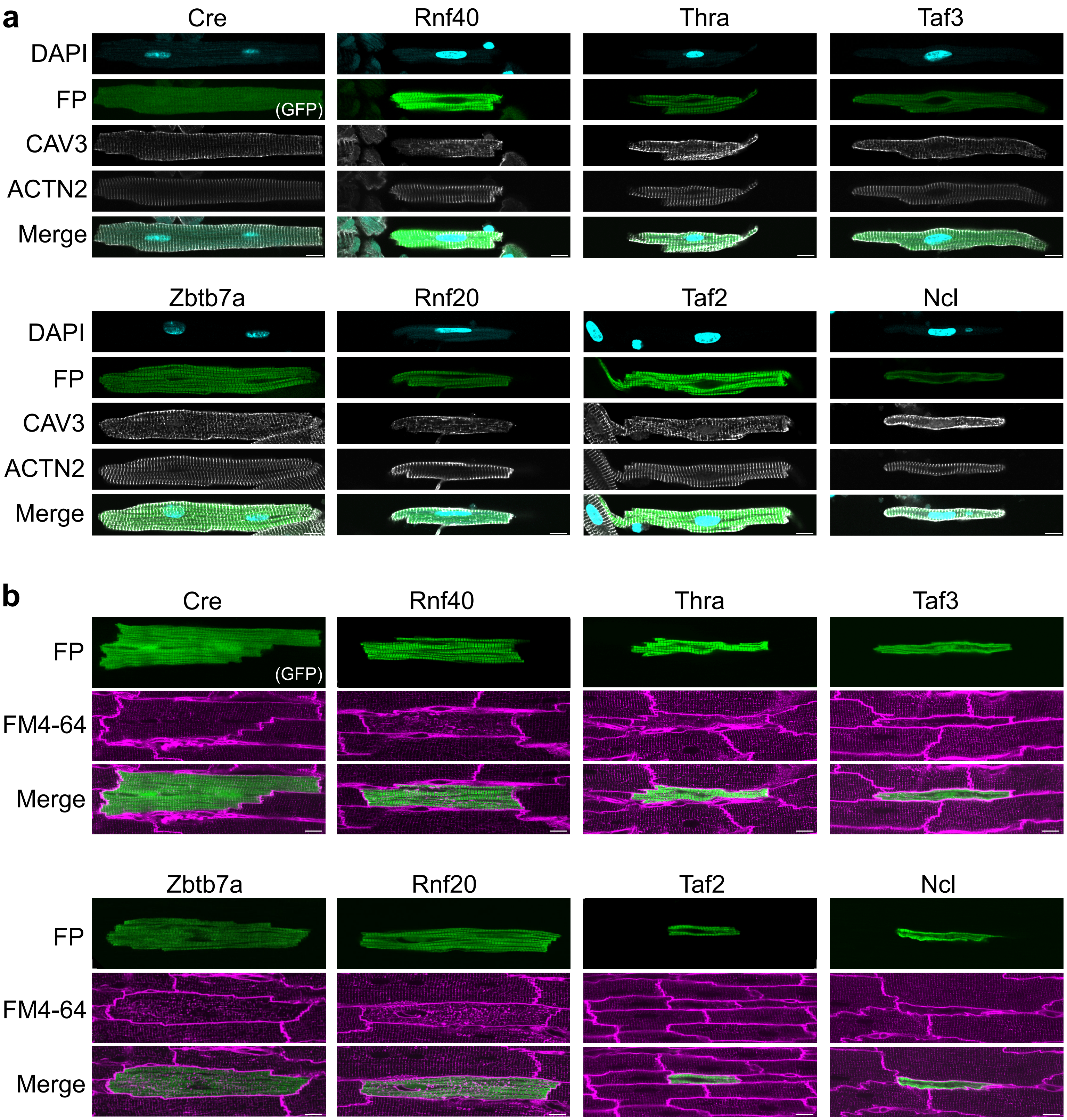

Suppl. Fig. 3. Validation of genetic screen. CASAAV vectors targeting each of the indicated factors, or AAV-Cre negative control, was delivered to neonatal mice at a dose that transduced a low percentage of cardiomyocytes. At P28, hearts were dissociated and isolated CMs were fixed and immunostained. Phenotype of fluorescent protein (FP) positive cells (GFP for AAV-Cre, YFP for CASAAV vectors) was examined. **a.** Confocal detection of FP, DAPI (nuclei), CAV3 immunostaining (T-tubules), and ACTN2 immunostaining (sarcomere Z-lines; n = 3 mice). **b.** Confocal detection of FP and FM4-64, which stains the sarcolemma and its invaginations, the T-tubules (n=3 mice). Scale bars = 10 μm. (continued)

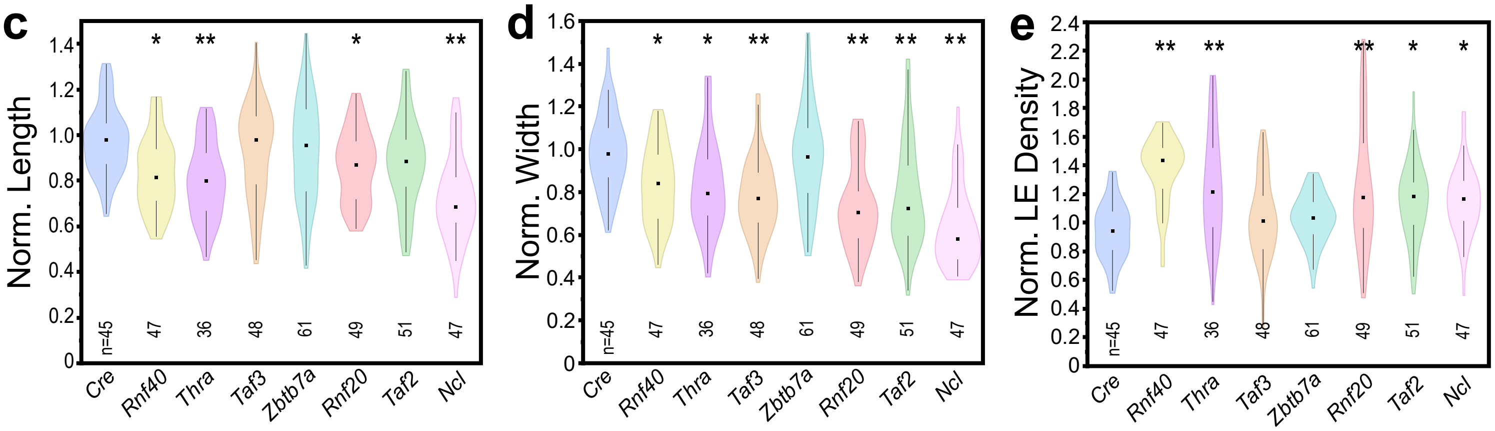

Suppl. Fig. 3, continued. c-d. Morphometric measurement of dissociated cardiomyocytes. Neonatal mice were treated with CASAAV targeting the indicated gene, or AAV-Cre control, and cardiomyocytes were dissociated at 4 weeks of life. **e.** Quantification of T-tubule longitudinal element (LE) density. Immature or stressed CMs feature higher LE density. Violin plots in c-e are formatted as described in Figure 2. n=3 mice for c-e, with CM number shown below each distribution. Dunnett's two-tailed t-test vs. Cre control: *$P<0.05$, **$P<0.001$. Source data are provided as a Source Data file.

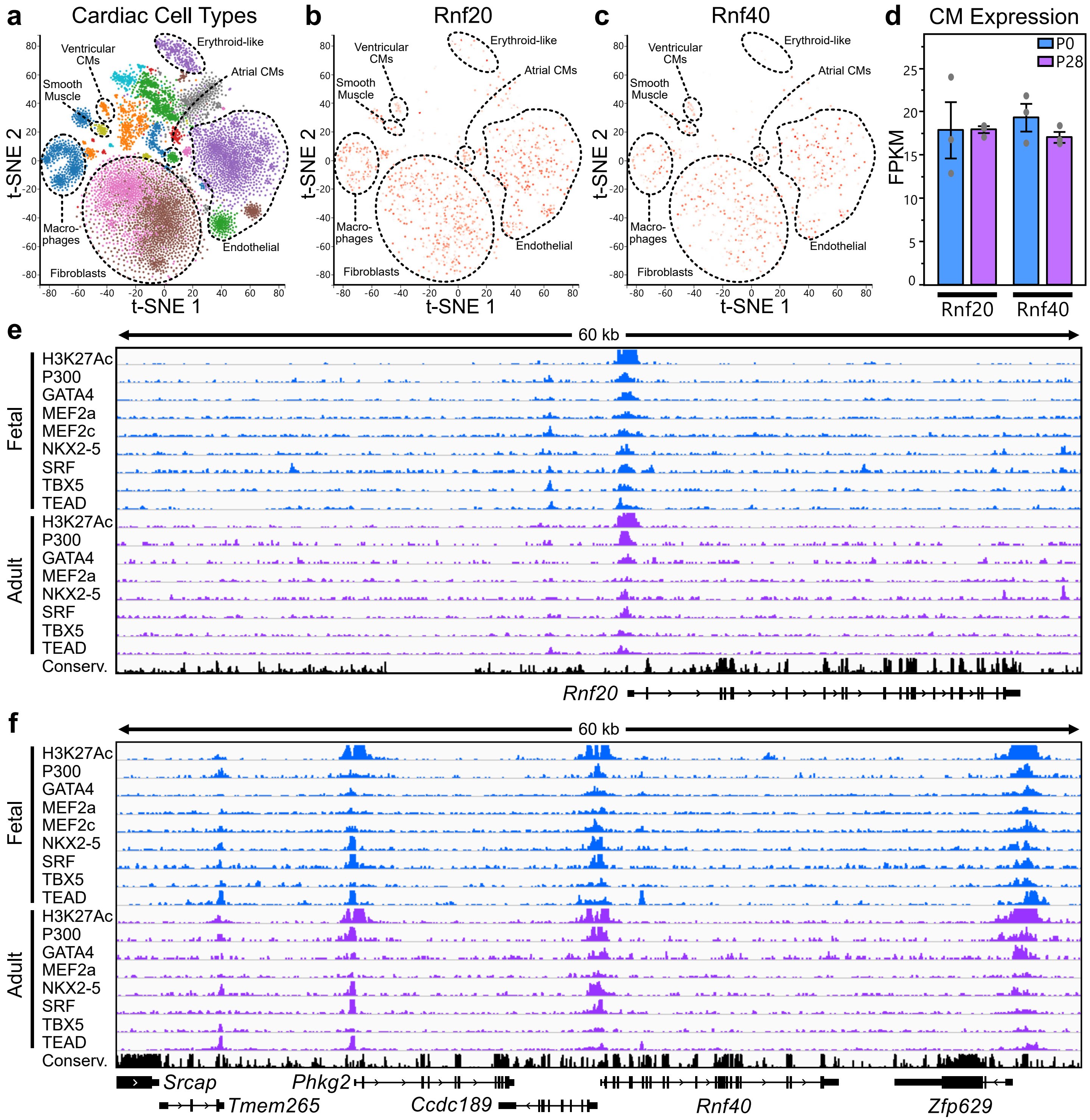

Suppl. Fig. 4. Cardiac expression profile and regulation of *Rnf20/40*. a. Single cell RNA-sequencing of the adult mouse heart; Panglao database SRA653146. Clustering identified 27 distinct cell populations. **b.** *Rnf20* expression within each population. Dot color intensity corresponds to expression strength. **c.** *Rnf40* expression within each population. **d.** Expression of *Rnf20* and *Rnf40* within newborn and adult ventricular CMs. Data are presented as mean values +/- SEM. **e.** Chromatin occupancy of enhancer markers and core cardiac transcription factors at the *Rnf20* locus, at fetal and adult stages, within heart whole apex tissue. **f.** Chromatin occupancy at the *Rnf40* locus. Source data are provided as a Source Data file.

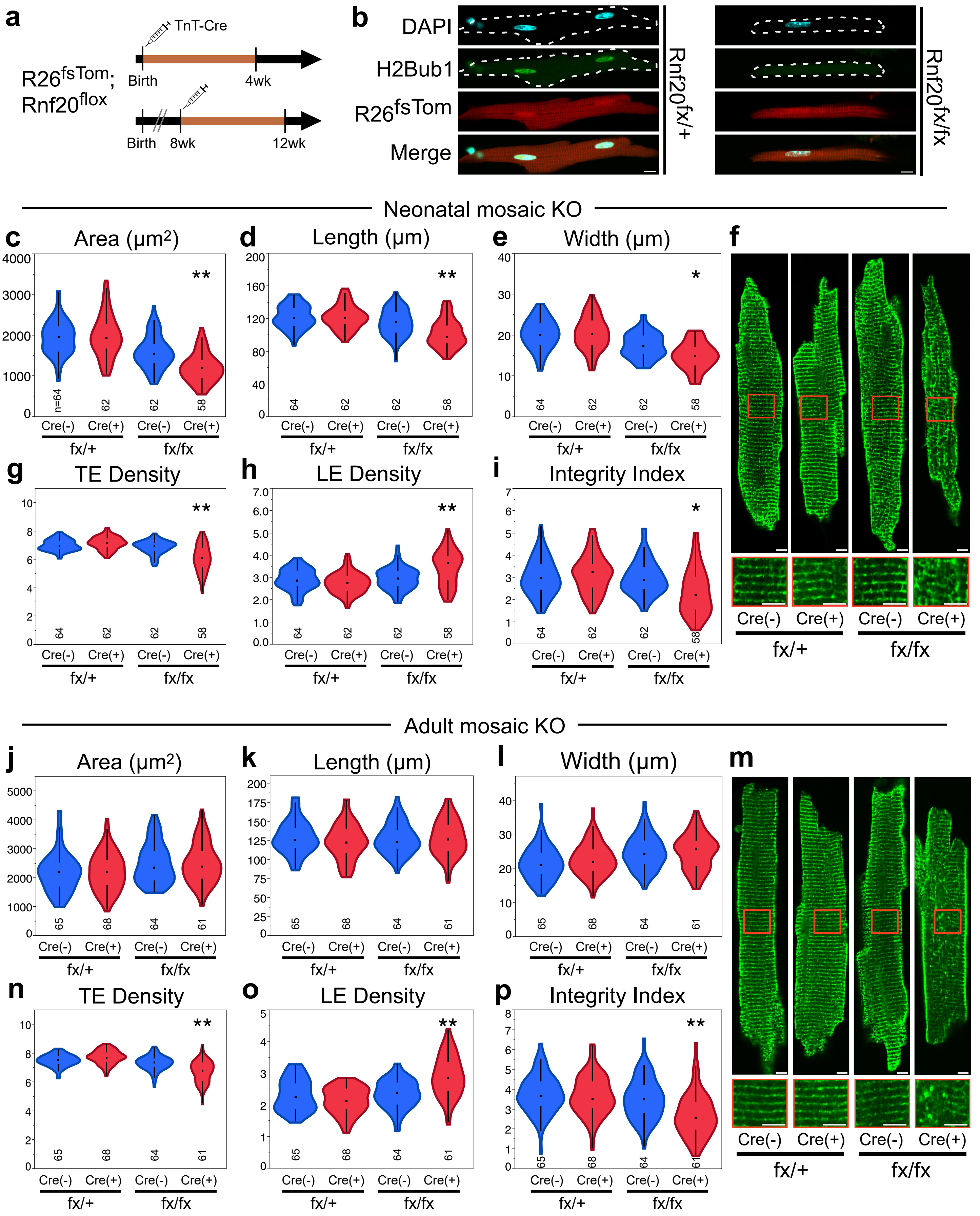

Suppl. Fig. 5. Mosaic inactivation of *Rnf20* at neonatal and adult stages using AAV-Cre and an *Rnf20* floxed allele. a. AAV9-TnT-Cre was delivered to neonatal or adult Rnf20^{flox} mice carrying a tdTomato Cre reporter allele (R26^{fsTom}), and CMs were dissociated and analyzed at 1 mo or 3 mo of age, respectively. (continued)

Suppl. Fig. 5, continued. Panels c-i show neonatal knockout; panels j-p show adult knockout. **b.** Immunostaining confirmed efficient depletion of H2Bub1 in Cre(+); Rnf20$^{fx/fx}$ CM nuclei (n=3 mice). Scale bars = 10 µm. **c-e.** Morphometric measurment of CMs dissociated at 4 weeks of age. Area and length *P*<0.0001; width *P*=0.0003. **f.** CAV3 immunostaining of CMs dissociated at 4 weeks of age. Scale bars = 5 µm. **g-i.** Quantification of T-tubule transverse element density (*P*<0.0001), longitudinal element density (*P*<0.0001), and integrity index (*P*=0.0003), respecitively. **j-l.** Morphometric measurment of 12-week dissociated CMs. **m.** CAV3 immunostaining of 12-week dissociated CMs. Scale bars = 5 µm. **n-p.** Quantification of T-tubule transverse element density, longitudinal element density, and integrity index, respecitively (*P*<0.0001 for all). Dunnett's two-tailed t-test vs. Cre(-); Rnf20$^{fx/fx}$: **P*<0.05, ***P*<0.001. Violin plots: shape indicates data distribution; point, median; whiskers, starts at quartile and extends 1.5 times the interquartile distance. Source data are provided as a Source Data file. For panels c-p n=3 mice each genotype; ~60 CMs each group, as indicated below distributions in each plot.

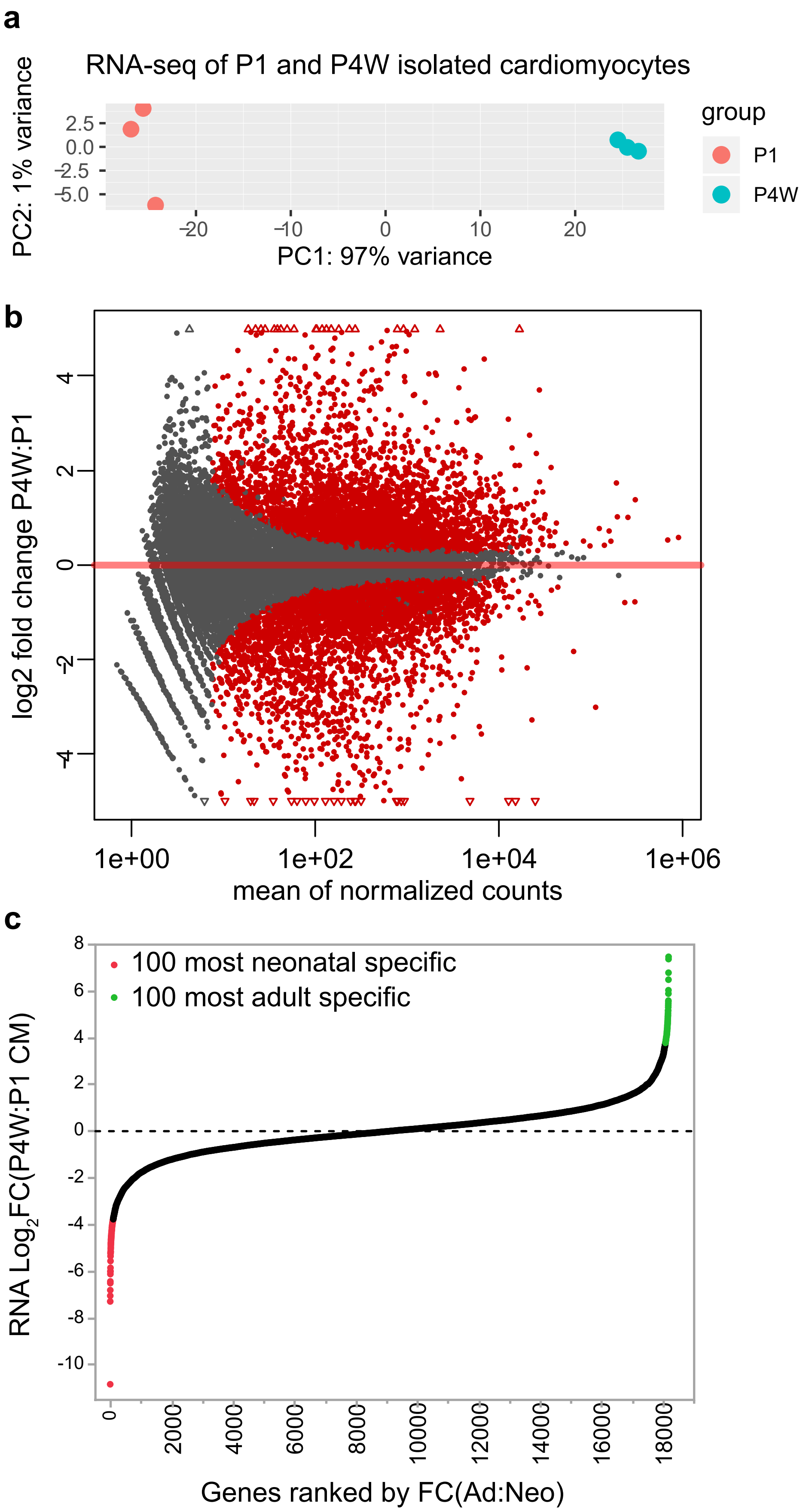

Suppl. Fig. 6. RNA-seq data from neonatal and 4-week-old cardiomyocytes. a. PCA plot showing separation of biological triplicate samples by age. **b.** MA plot of differential gene expression analysis. Red, P_{Adj}<0.05. **c.** P4W:P1 ratio highlighting the 100 most neonatal or adult specific genes. Source data are provided as a Source Data file.

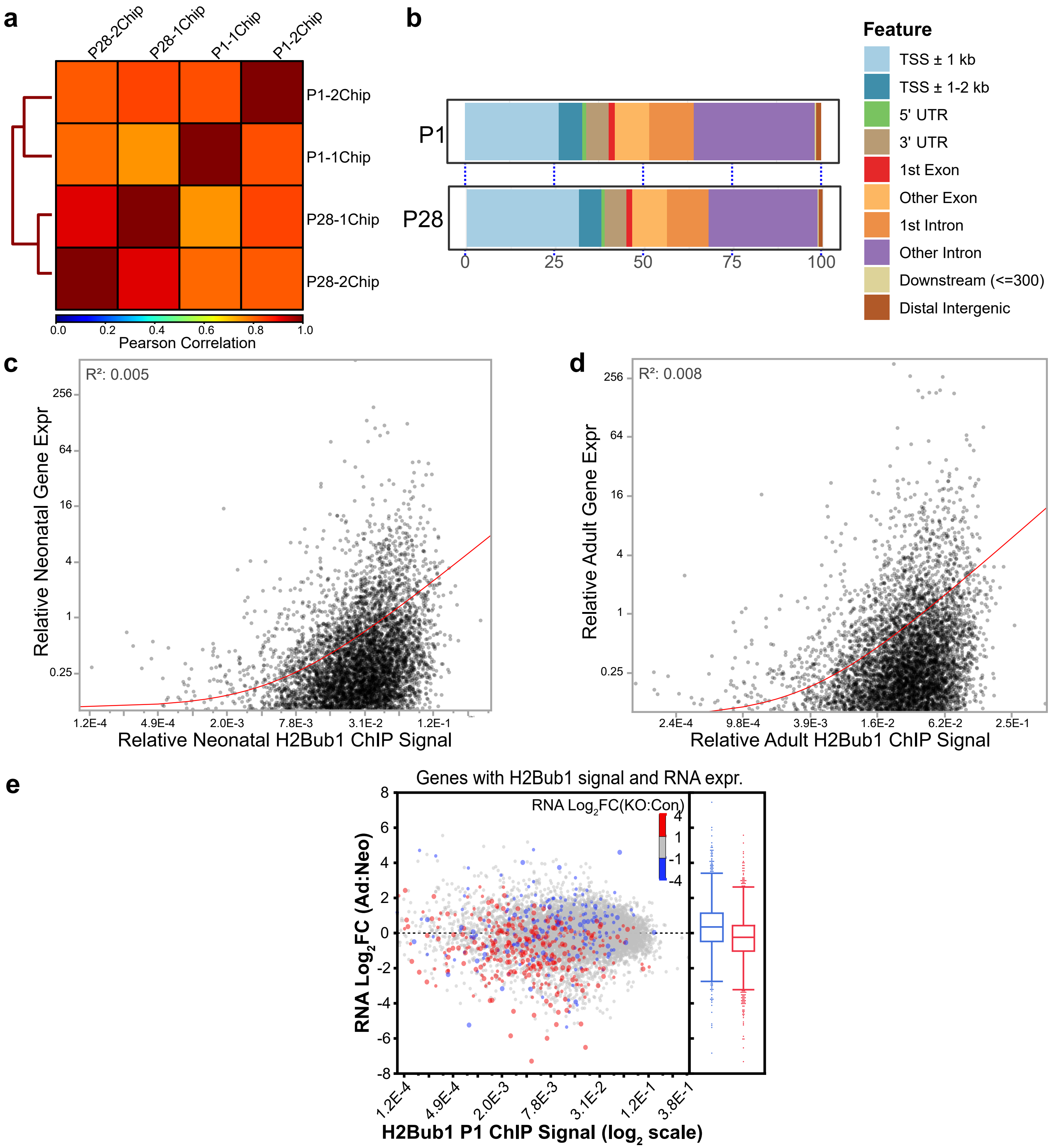

Suppl. Fig. 7. H2Bub1 ChIP-seq. a. Correlation between biological duplicate H2Bub1 ChIP-seq samples prepared from P1 (neonatal) or P28 (adult) heart apexes. Hierarchical clustering by Pearson correlation. **b.** Location of H2Bub1 broad peaks, called by MACS2, with respect to genome annotations. The vast majority of reads fell within gene bodies. **c-d.** Relationship between gene expression and H2Bub1 ChIP signal in neonatal or adult stages. ChIP signal was the input subtracted ChIP, normalized for library depth, composition, and gene length. More active genes tended to have higher H2Bub1 signal, but the correlation was poor. **e.** Maturational change in RNA expression versus H2Bub1 P1 ChIP signal. ChIP signal was input substracted and normalized for sequencing depth, library composition, and gene length. Points are colored by the change in RNA expression in RNF20/40-depleted CMs versus control CMs. Genes upregulated in RNF20/40 depletion (red) generally were downregulated during maturation and had moderate H2Bub1 occupancy. Center lines in box plots indicate the median, while boxes show 25th and 75th percentiles. Whiskers denote the maximum observation within the 75th percentile + 1.5 times the interquartile range, or the minimum observation within the 25th percentile - 1.5 times the interquartile range. For genes with Log_2FC(KO:Con) from -1 to -4 (blue box-plot) n=1053; from 1 to 4 (red) n=2128. (continued)

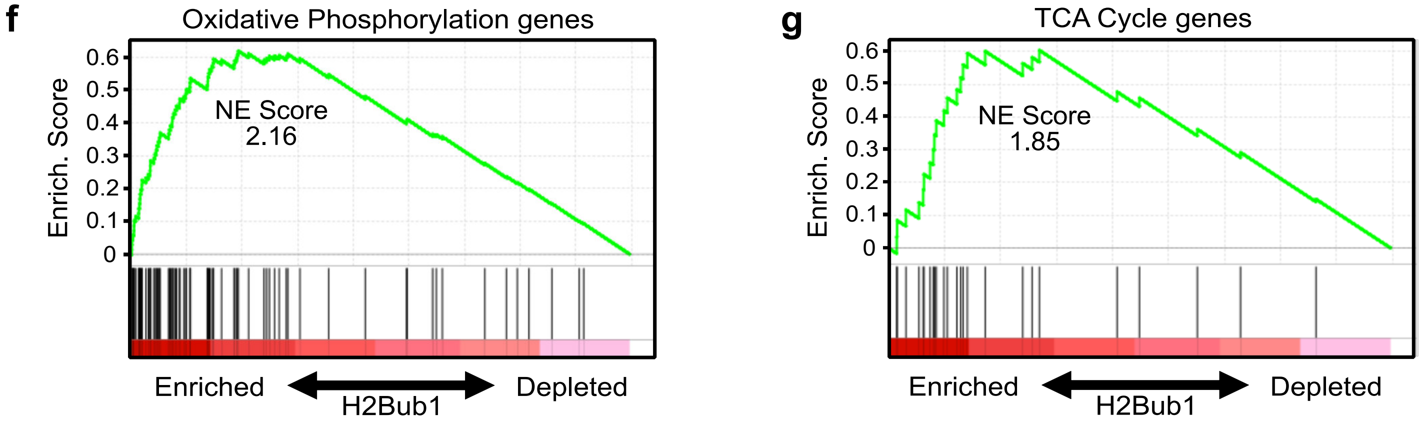

Suppl. Fig. 7. continued. f-g. Gene set enrichment analysis of genes ranked by P1 H2Bub1 signal shows that oxidative phosphorylation and TCA cycle genes were preferentially marked. Source data are provided as a Source Data file.

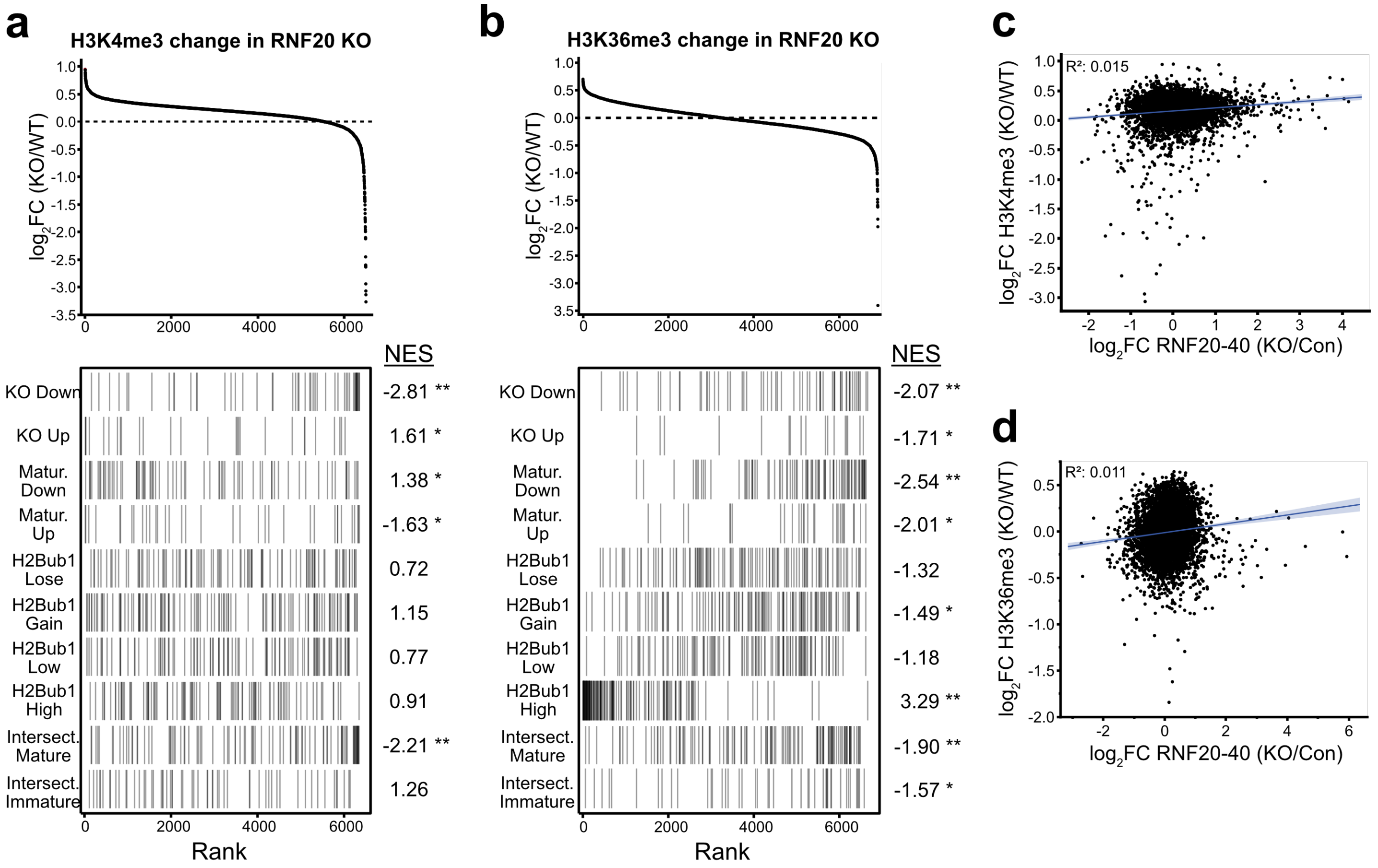

Suppl. Fig. 8. Gene set enrichment analysis of changes in H3K4me3 or H3K36me3 signal upon *Rnf20* KO. Wildtype or *Rnf20*$^{fx/fx}$ newborn pups were injected with a high dose of AAV9-TnT-Cre. Heart apexes were collected at P7 and ChIP-sequencing conducted for H3K4me3 and H3K36me3. ChIP signal was quantified, marked genes were ranked by change in ChIP signal, and the relationship between ChIP signal and various gene sets of interest was examined by GSEA. Gene sets comprised of 200 genes were defined by: the genes most downregulated or upregulated in the *Rnf20/40* mosaic KO ("KO Down" and "KO Up", respectively); the genes displaying the largest loss or gain in H2Bub1 during maturation ("Matur. Down" and "Matur. Up", respectively); the genes that had the lowest or highest absolute levels of H2Bub1 at P1 ("H2Bub1 Low" and "H2Bub1 High", respectively). Gene sets consisting of the previously identified *Rnf20/40*-regulated maturation genes were also included (see Fig. 4g,h). The gene set "Intersect. Mature" contained the genes that are upregulated during maturation, gain H2Bub1 during maturation, and are downregulated in the *Rnf20/40* KO. The set "Intersect. Immature" contains genes that are downregulated during maturation, lose H2Bub1 during maturation, and are upregulated in *Rnf20/40* KO. **a-b.** H3K4me3 signal at each TSS or H3K36me3 signal over each gene body was quantified in the wild type and *Rnf20* KO condition, and genes were ranked by change in signal (upper panel). For each gene set the distribution of marked genes within the ranked list was analyzed by GSEA (lower panel). NES = normalized enrichment score. *P<0.05, **P<0.001. **c.** Scatterplot showing relationship between change in H3K4me3 in the RNF20 KO and change in gene expression in the RNF20/40 KO. **d.** Scatterplot showing relationship between change in H3K36me3 in the RNF20 KO and change in gene expression in the RNF20/40 KO. Best fit line with shading of 95% confidence interval is shown. Source data are provided as a Source Data file.

Gene	Geneset	FPKM:	P0_CM_1	P0_CM_3	P0_CM_2	P4W_CM_	P4W_CM_	P4W_CM_3	DESeq2:	baseMean	log2FoldCh	lfcSE	Wald stat	Wald pVal	Corrected pVal	maxFPKM(
Tnni1	Neonatal Specific		574.5256	688.3319	868.6538	0.193776	0.52575	0.4285389		15168.36	-10.8901	0.325465	-33.46	1.84E-245	2.63E-241	868.6538
Bex4	Neonatal Specific		56.07221	72.19118	98.79191	0.228701	0.282048	0.9195898		275.3845	-7.27916	0.632545	-11.5077	1.21E-30	4.17E-29	197.2064
Cdh3	Neonatal Specific		1.022736	0.945976	1.394254	0.023766	0.014655	0		78.89993	-6.46482	0.880905	-7.33883	2.15E-13	2.39E-12	49.93036
Aldoc	Neonatal Specific		1.393525	2.064218	2.257525	0.057907	0	0.0291052		55.23868	-5.95673	0.897565	-6.63654	3.21E-11	2.89E-10	14.25238
Hist1h2bp	Neonatal Specific		0.961166	0.35127	0.835948	0	0	0.0304709		19.81337	-5.93001	1.29754	-4.57019	4.87E-06	2.24E-05	159.5305
Lars2	Neonatal Specific		2904.964	580.5579	3783.811	40.59623	41.75453	69.681177		12748.73	-5.57922	0.755157	-7.38816	1.49E-13	1.69E-12	13.07093
Hmgcs2	Neonatal Specific		291.8549	236.6021	369.386	7.068659	8.911243	5.7371515		4840.888	-5.37538	0.195102	-27.5517	4.23E-167	1.01E-163	9.99391
Prrx2	Neonatal Specific		0.489837	0.245175	0.487989	0	0	0		6.234993	-5.24691	1.462716	-3.5871	3.34E-04	NA	3.71689
Vash2	Neonatal Specific		29.14933	18.91196	30.64982	0.54863	0.45107	1.1030007		243.616	-5.19845	0.384076	-13.535	9.72E-42	5.43E-40	34.89909
Cacng6	Neonatal Specific		0.585428	0.701805	0.907231	0.057439	0	0		21.6132	-5.1741	1.128865	-4.58345	4.57E-06	2.10E-05	26.16978
Tubb2b	Neonatal Specific		6.407685	4.98089	6.548748	0.176819	0.118944	0.1939028		315.9848	-5.17294	0.328326	-15.7555	6.30E-56	6.38E-54	1.741781
Egr2	Neonatal Specific		4.581604	4.811977	5.736582	0.066323	0.163588	0.2000108		129.3375	-5.15254	0.476083	-10.8228	2.69E-27	7.76E-26	997.7668
Ccnb1	Neonatal Specific		159.6051	117.3375	235.6231	5.275711	3.71791	5.3033163		192.6916	-5.14825	0.427716	-12.0366	2.28E-33	8.89E-32	21.2162
Fosb	Neonatal Specific		30.36986	29.22128	40.59346	0.901131	0.977972	1.123249		789.0876	-5.05984	0.237937	-21.2654	2.37E-100	8.70E-98	4.541346
Cks2	Neonatal Specific		4.161265	2.794233	5.254468	0.204141	0.08392	0.1368063		51.18094	-4.81043	0.686645	-7.0057	2.46E-12	2.47E-11	4.828389
Capn6	Neonatal Specific		50.68146	63.80187	66.73182	2.054259	2.895364	2.3600093		177.0759	-4.65025	0.367716	-12.6463	1.17E-36	5.24E-35	20.8604
Dlgap5	Neonatal Specific		14.93059	17.07846	19.7281	0.415906	0.820676	0.919782		178.5908	-4.59913	0.364006	-12.6348	1.36E-36	6.05E-35	1.784512
Cdk1	Neonatal Specific		19.33968	30.00602	25.36203	1.179907	0.842448	1.1236561		345.5678	-4.55922	0.289259	-15.7617	5.71E-56	5.83E-54	1.868691
Hmmr	Neonatal Specific		46.37276	54.63545	57.32066	3.284377	1.175952	2.5560484		430.1785	-4.46526	0.259162	-17.2296	1.59E-66	2.21E-64	25.76382
Igf2bp3	Neonatal Specific		37.72012	41.7614	45.85958	1.868776	1.491272	2.3205671		328.5362	-4.45035	0.272373	-16.3392	5.19E-60	5.99E-58	5.278893
Zfp618	Neonatal Specific		17.79526	20.585	20.81189	1.300455	0	1.30726		78.5944	-4.4291	0.501111	-8.83857	9.69E-19	1.69E-17	45.10958
Socs3	Neonatal Specific		99.37674	74.74804	114.6913	4.692681	5.125906	4.0433447		622.2661	-4.38669	0.236026	-18.5856	4.20E-77	8.46E-75	3.746291
Marcksl1	Neonatal Specific		9.823917	12.457	10.21291	0.662578	0.514491	0.3946932		382.6407	-4.36937	0.259543	-16.8348	1.36E-63	1.76E-61	2.280366
Ly6g6d	Neonatal Specific		0.324275	0.370989	0.287157	0	0	0		3.30996	-4.34066	1.650677	-2.62962	8.55E-03	NA	4.966301
Snca	Neonatal Specific		3.598248	10.28625	4.92607	0.438259	0.360326	0.1468508		74.55341	-4.33079	0.559898	-7.73496	1.03E-14	1.30E-13	14.59936
Plagl1	Neonatal Specific		2348.254	2392.896	2711.568	97.99998	157.484	123.88728		1453.931	-4.30694	0.181185	-23.771	6.67E-125	4.33E-122	21.94428
Birc5	Neonatal Specific		41.23591	51.92663	57.95268	2.275797	2.606182	2.7779276		269.2311	-4.3069	0.293078	-14.6954	6.90E-49	5.48E-47	5381.049
Fbn2	Neonatal Specific		34.43668	38.85827	38.94973	1.916118	2.363078	1.579438		849.4897	-4.27227	0.1949	-21.9203	1.66E-106	7.67E-104	23.20323
Cenpm	Neonatal Specific		1.384659	2.427963	2.293652	0.143847	0.141921	0.05784		61.79711	-4.1776	0.54271	-7.69768	1.39E-14	1.72E-13	2.444613
Nts	Neonatal Specific		13.90397	14.22191	14.25238	0.473737	0.973738	1.0318017		156.602	-4.10988	0.34025	-12.079	1.36E-33	5.37E-32	16.01097
Anln	Neonatal Specific		66.77463	76.48954	85.15896	4.052051	4.652609	4.6350814		476.9369	-4.10255	0.226507	-18.1123	2.55E-73	4.50E-71	2.026302
Nek2	Neonatal Specific		4.180718	4.106266	5.213794	0.43432	0.252062	0.1027277		154.1758	-4.094	0.357881	-11.4395	2.65E-30	9.00E-29	1.53587
Kif4	Neonatal Specific		16.63041	16.46674	21.35828	1.350867	0.913599	0.9636963		364.8796	-4.06771	0.252997	-16.0781	3.64E-58	3.94E-56	13.00353
Bmp7	Neonatal Specific		18.60845	17.04049	26.15755	0.957198	1.652669	1.1546485		283.2365	-4.05858	0.289622	-14.0134	1.29E-44	8.21E-43	188.2504
Igf2bp1	Neonatal Specific		8.938505	7.705128	9.275824	0.822189	0.101398	0.5785436		91.93255	-4.04834	0.432002	-9.37111	7.18E-21	1.43E-19	72.03134
Fam83d	Neonatal Specific		1.896775	1.841837	2.352861	0.026586	0.13115	0.2138009		67.00851	-4.0432	0.500559	-8.07737	6.62E-16	9.14E-15	74.85996
Cited1	Neonatal Specific		22.0505	27.90368	37.87931	1.915216	2.099526	1.3904493		241.5456	-4.03611	0.308951	-13.0639	5.29E-39	2.64E-37	16.74431
Ccdc113	Neonatal Specific		3.298674	4.600414	3.370495	0	0.414497	0.3378561		29.57868	-3.96257	0.719462	-5.50769	3.64E-08	2.28E-07	641.7685
Ckap2l	Neonatal Specific		22.61607	35.41378	30.67845	1.886357	1.314908	2.555785		317.4861	-3.93068	0.27523	-14.2815	2.86E-46	2.01E-44	8.400467
Tgif2	Neonatal Specific		2.02085	1.798198	2.705608	0.127463	0.157195	0.1494849		90.29212	-3.91812	0.426886	-9.17837	4.38E-20	8.26E-19	2.912205
Hey2	Neonatal Specific		32.73412	30.04977	39.472	2.16717	2.41987	2.2079495		1026.399	-3.91492	0.183941	-21.2836	1.61E-100	6.06E-98	21.71935
Pdk3	Neonatal Specific		11.6621	8.855292	12.6571	0.621171	0.826152	0.7835878		801.2659	-3.89908	0.205871	-18.9394	5.40E-80	1.21E-77	7.170078
Bub1b	Neonatal Specific		18.16116	22.28248	19.83922	1.030486	1.429719	1.6833018		413.6005	-3.86668	0.231545	-16.6995	1.32E-62	1.64E-60	110.9342
Col18a1	Neonatal Specific		14.06918	11.0996	14.66386	0.755859	0.932174	1.0499254		853.8463	-3.86346	0.196623	-19.6491	5.89E-86	1.50E-83	1.364212
Acot1	Neonatal Specific		56.15077	57.34966	73.7225	5.417175	4.165679	3.3313776		865.6095	-3.85681	0.204037	-18.9025	1.09E-79	2.39E-77	15.93867
Racgap1	Neonatal Specific		14.94402	14.6575	17.45487	0.856889	1.637994	0.8613729		324.3344	-3.83895	0.255177	-15.0443	3.76E-51	3.36E-49	3.693709
Mdk	Neonatal Specific		2.87038	5.134861	4.765922	0.316831	0.390736	0.2123257		35.7338	-3.82634	0.641608	-5.96367	2.47E-09	1.80E-08	2.678346
Ndc80	Neonatal Specific		16.42744	25.70023	22.05869	1.79347	1.040857	1.6968041		179.5438	-3.80842	0.321567	-11.8433	2.33E-32	8.70E-31	144.7973
Cdc25b	Neonatal Specific		5.585741	6.390408	6.403791	0.308275	0.597433	0.4426976		123.4805	-3.79581	0.354625	-10.7037	9.78E-27	2.73E-25	20.0072
Hyi	Neonatal Specific		0.944531	1.219363	1.713968	0.027346	0.033725	0.2199129		41.92053	-3.75056	0.607806	-6.17064	6.80E-10	5.30E-09	172.0349
Acsl3	Neonatal Specific		69.76025	127.3964	93.53123	6.398202	7.772014	7.9307952		1792.228	-3.71925	0.206869	-17.9787	2.86E-72	4.76E-70	42.45743
Mest	Neonatal Specific		24.67335	22.02434	33.40144	1.860603	2.3796	1.9396105		1035.343	-3.70202	0.200097	-18.5011	2.02E-76	4.02E-74	28.57188
Aurka	Neonatal Specific		8.95576	5.882468	11.0161	0.701916	0.461678	0.7996668		163.8604	-3.70064	0.343731	-10.7661	4.98E-27	1.42E-25	36.81408
Mdfi	Neonatal Specific		1.737085	2.392151	1.922815	0.073043	0.180163	0.2202762		24.62547	-3.69314	0.732142	-5.0443	4.55E-07	2.46E-06	11.97904
Tcf19	Neonatal Specific		9.192699	7.933858	9.884855	0.366202	1.053788	0.736236		65.86432	-3.68195	0.462165	-7.96675	1.63E-15	2.18E-14	146.3455
Mmp14	Neonatal Specific		14.57111	47.91746	21.08452	1.58679	2.977938	2.0112042		360.1784	-3.67311	0.596284	-6.16	7.27E-10	5.65E-09	8.11263
Ckap2	Neonatal Specific		22.93189	26.9007	32.22902	2.310772	2.399825	1.8338397		200.8205	-3.65807	0.291621	-12.5439	4.29E-36	1.88E-34	1.186933
Nuf2	Neonatal Specific		2.762427	4.139599	3.45726	0.231764	0.414448	0.1980305		266.4934	-3.64699	0.280298	-13.0111	1.06E-38	5.09E-37	92.4682
Klc3	Neonatal Specific		0.065236	0.048374	0.028884	0	0	0		2.045508	-3.63227	1.89227	-1.91953	5.49E-02	NA	9.089701
Dusp2	Neonatal Specific		1.102317	1.219795	0.938597	0.149751	0.061561	0.0501783		19.44969	-3.61614	0.806238	-4.4852	7.28E-06	3.24E-05	1.585982
Zfp184	Neonatal Specific		2.406879	2.566995	2.531956	0.120588	0.223075	0.272742		74.26442	-3.61439	0.427144	-8.46175	2.63E-17	4.18E-16	1.343088
Tro	Neonatal Specific		12.59675	9.927662	11.46853	0.516734	1.106835	1.1755691		372.8974	-3.61109	0.247464	-14.5924	3.14E-48	2.44E-46	114.4644
Rrm2	Neonatal Specific		13.22393	13.90198	17.36017	1.492754	1.12833	1.0165117		275.4739	-3.60968	0.258111	-13.985	1.92E-44	1.21E-42	129.4328
Pafah1b3	Neonatal Specific		3.962852	4.100879	4.847962	0.207368	0.639349	0.2605664		74.53884	-3.5998	0.43476	-8.27995	1.23E-16	1.85E-15	449.768
Myl9	Neonatal Specific		243.4739	245.25	309.2451	27.06165	22.56858	16.98345		6446.315	-3.58261	0.175334	-20.4331	8.49E-93	2.38E-90	71.9314
Ect2	Neonatal Specific		4.09752	3.019217	5.196656	0.456562	0.337837	0.2753705		161.5985	-3.52132	0.328932	-10.7053	9.61E-27	2.69E-25	5.651555
Arhgap11a	Neonatal Specific		35.24916	21.4606	38.3086	3.276447	2.755038	2.2456301		191.1829	-3.51885	0.315822	-11.1419	7.85E-29	2.46E-27	27.40307
Prc1	Neonatal Specific		38.2112	50.90058	54.58296	4.552469	4.29336	3.9706044		644.4	-3.48903	0.203932	-17.1088	1.28E-65	1.70E-63	1889.375
Fignl1	Neonatal Specific		13.85262	10.68782	15.08359	1.108672	1.823047	0.7429821		161.4773	-3.46008	0.31803	-10.8797	1.44E-27	4.22E-26	5.361222
Trim10	Neonatal Specific		0.714183	1.668177	1.185816	0.157662	0	0.1584872		6.791041	-3.4385	1.305554	-2.63375	8.44E-03	NA	37.04461
Ddc	Neonatal Specific		63.63272	88.11627	100.7688	7.156718	8.349033	7.9071017		1179.661	-3.4343	0.193371	-17.7601	1.44E-70	2.29E-68	30.98759
Dynlt1a	Neonatal Specific		0.610085	1.04502	1.017476	0.07183	0.088586	0.0902576		44.8712	-3.42878	0.534295	-6.41738	1.39E-10	1.16E-09	63.57048
Hist1h1a	Neonatal Specific		41.23982	62.68919	62.51955	3.694397	6.834248	5.5705922		190.6818	-3.39091	0.300157	-11.2971	1.36E-29	4.46E-28	1.510379
Hist1h3d	Neonatal Specific		0.180395	0.066883	0.226307	0	0.026194	0.0213504		13.52642	-3.35978	0.967296	-3.47337	5.14E-04	0.001656996	10.55956
Trip13	Neonatal Specific		1.967307	2.235855	2.475192	0.2064	0.127273	0.3112201		58.68044	-3.3479	0.455361	-7.3522	1.95E-13	2.17E-12	10.80482
Mcm10	Neonatal Specific		13.28292	12.94099	14.38116	2.054764	0.633517	1.2048843		71.63252	-3.34718	0.417864	-8.01021	1.15E-15	1.55E-14	15.20269
Cep55	Neonatal Specific		11.27824	15.50718	17.71681	1.377319	1.306614	1.7040323		127.1975	-3.34135	0.33704	-9.91382	3.63E-23	8.39E-22	3.695643
Insc	Neonatal Specific		0.510362	0.87988	0.553631	0.045067	0.083369	0.0679538		26.18117	-3.3348	0.67228	-4.96044	7.03E-07	3.69E-06	16.40636
Col23a1	Neonatal Specific		4.356103	4.310936	4.436844	0.35102	0.36075	0.5880941		135.7292	-3.32291	0.31214	-10.6456	1.83E-26	5.03E-25	41.06192
Brca1	Neonatal Specific		20.54761	21.64196	22.31134	1.604594	1.978888	2.8537519		158.4186	-3.32137	0.294316	-11.285	1.56E-29	5.09E-28	10.93702
Tceal7	Neonatal Specific		60.6912	71.4627	61.85223	7.084059	7.596968	4.9538275		191.1958	-3.31605	0.27425	-12.0913	1.17E-33	4.66E-32	5.247248
Mst1r	Neonatal Specific		1.218704	0.963938	0.419691	0	0.147465	0.1201988		6.616009	-3.31584	1.31451	-2.52249	1.17E-02	NA	64.91766
Nusap1	Neonatal Specific		15.06233	18.83251	18.6707	1.985159	2.028529	1.3683748		281.6154	-3.29772	0.243044	-13.5684	6.17E-42	3.47E-40	10.37621
Mki67	Neonatal Specific		43.60745	45.32082	49.54691	4.1767	5.547201	4.4645265		2226.603	-3.29106	0.15341	-21.4528	4.30E-102	1.66E-99	11.82078
Ncam1	Neonatal Specific		31.46438	42.83597	43.68293	2.897415	5.206776	4.0568029		1733.367	-3.28398	0.200175	-16.4056	1.75E-60	2.08E-58	11.83967
Dscc1	Neonatal Specific		39.08696	41.42965	45.55671	3.045454	6.259746	4.0818528		37.86764	-3.26781	0.545418	-5.99139	2.08E-09	1.53E-08	4.436508
Cdca5	Neonatal Specific		1.792002	1.507903	2.242335	0.4128	0.084849	0.06916		24.47559	-3.25054	0.68978	-4.71243	2.45E-06	1.19E-05	30.79644
Kif23	Neonatal Specific		9.08286	12.02606	11.33009	0.92544	1.474195	1.0853298		256.8002	-3.23683	0.252336	-12.8275	1.15E-37	5.39E-36	6.959299
Rbp1	Neonatal Specific		7.786395	9.580159	11.76855	0.679077	1.360907	1.1092748		104.8362	-3.23424	0.358603	-9.01899	1.90E-19	3.46E-18	4.606932
Neil3	Neonatal Specific		9.20682	6.92584	8.608896	0.748811	1.187333	0.7527288		70.68168	-3.22068	0.411497	-7.82674	5.01E-15	6.45E-14	34.84493
Heph	Neonatal Specific		12.6449	13.78191	16.41476	1.5598	1.313709	1.835663		344.5247	-3.17895	0.224959	-14.1313	2.44E-45	1.62E-43	2.844562
Aif1l	Neonatal Specific		1.995783	2.198724	2.67396	0.259393	0.206994	0.2914268		137.7691	-3.17212	0.308846	-10.2709	9.53E-25	2.40E-23	5.786465
Tmem178	Neonatal Specific		0.556841	0.348182	0.406476	0	0.098336	0.053436		15.17525	-3.17107	0.852418	-3.72008	1.99E-04	0.000695209	954.3238
Dsn1	Neonatal Specific		7.733528	7.858715	9.279676	0.565714	1.096346	1.1373481		94.37987	-3.16557	0.359547	-8.80434	1.32E-18	2.28E-17	2043.44

Sox4	Neonatal Specific	16.22647	78.89013	27.51235	4.00073	4.641573	5.0941085	1268.372	-3.15845	0.688275	-4.58893	4.46E-06	2.06E-05	17.92682
Nipal4	Neonatal Specific	3.80221	3.955848	3.910894	0.516536	0.127405	0.6230866	34.51572	-3.15541	0.561086	-5.62374	1.87E-08	1.22E-07	16.44693
Prim1	Neonatal Specific	2.325963	2.466552	3.219569	0.279172	0.270515	0.3608132	123.3376	-3.13424	0.324014	-9.67316	3.92E-22	8.51E-21	17.28702
Spag5	Neonatal Specific	25.5438	23.92924	31.21024	2.513579	3.589365	3.1916615	187.4757	-3.12866	0.273499	-11.4394	2.66E-30	9.00E-29	40.5637
Gins2	Neonatal Specific	3.38353	4.787349	3.806033	0.376836	0.464739	0.5411547	68.40504	-3.12042	0.41154	-7.58231	3.39E-14	4.05E-13	2.848698
Mastl	Neonatal Specific	34.39504	27.71549	34.08851	1.880621	4.638602	4.7261528	157.482	-3.10957	0.30315	-10.2575	1.09E-24	2.75E-23	2.485129
Irf7	Adult Specific	2.678187	3.614384	3.24123	15.13557	24.11044	28.210713	134.604	2.804576	0.281824	9.951509	2.48E-23	5.82E-22	13.63118
Cyp4b1	Adult Specific	0.38528	0.421397	0.433583	2.721728	3.21675	2.8499685	97.50337	2.812768	0.275914	10.19437	2.10E-24	5.15E-23	2.763423
Srrm4	Adult Specific	0.696376	0.318199	0.216667	2.825329	3.033457	2.4391559	79.02305	2.830813	0.369363	7.664035	1.80E-14	2.20E-13	6.809683
Map2k3	Adult Specific	1.021579	0.99795	1.371718	8.844403	8.803378	6.6630836	389.3759	2.83321	0.201223	14.07997	5.04E-45	3.29E-43	24.52423
Metrn	Adult Specific	0.187596	0.27376	0.249184	1.775805	1.944882	1.4653781	122.6478	2.838021	0.264926	10.7125	8.89E-27	2.50E-25	8.288174
Kcnj8	Adult Specific	60.8377	99.24702	103.7573	553.1357	618.3628	768.04142	152.6234	2.843552	0.259759	10.94688	6.88E-28	2.05E-26	1.562165
Asap3	Adult Specific	0.510162	0.610774	0.496944	3.800507	4.485001	3.458115	224.8278	2.851354	0.214625	13.28527	2.82E-40	1.49E-38	38.8404
Lyz2	Adult Specific	2.108655	2.74748	2.467491	12.76811	17.3867	24.065469	127.5493	2.873101	0.286562	10.02611	1.17E-23	2.78E-22	73.43508
Osgin1	Adult Specific	0.121929	0.271238	0.026993	2.045684	0.53113	0.5411547	9.058096	2.883935	0.946074	3.048318	2.30E-03	0.006365254	48.84921
Mndal	Adult Specific	1.137578	2.099466	1.193916	7.366491	11.10368	14.884872	139.976	2.890965	0.311002	9.295656	1.46E-20	2.85E-19	43.32211
Cd300ld	Adult Specific	0.116027	0.019358	0.012843	0.332982	0.252711	0.3604726	11.77726	2.891064	0.822414	3.515339	4.39E-04	0.001430755	1.256816
Apol10b	Adult Specific	0.436156	0.089562	0.014855	0.948039	1.242256	1.3699369	19.07704	2.896072	0.743081	3.897383	9.72E-05	0.000359438	10095.22
Kcnd3	Adult Specific	4.159477	3.38395	3.814917	28.64911	30.54334	25.317821	251.8118	2.905798	0.202699	14.33552	1.31E-46	9.53E-45	2.89488
Asb14	Adult Specific	5.84305	6.288945	6.86283	55.73622	45.18993	41.989842	722.2592	2.906864	0.168798	17.22098	1.85E-66	2.54E-64	3.039694
AI593442	Adult Specific	0.828954	0.352047	0.083417	2.462166	3.528906	2.9433025	42.24558	2.925844	0.531447	5.505426	3.68E-08	2.30E-07	7.352484
Rsad2	Adult Specific	3.403343	9.137326	5.559912	36.89229	52.14369	52.294487	212.034	2.930725	0.284531	10.30021	7.03E-25	1.78E-23	1.09655
Enho	Adult Specific	0.178075	0.396139	0.525641	2.463534	3.619967	2.8979434	53.01565	2.953458	0.396058	7.457134	8.84E-14	1.02E-12	1.368923
Nlrc5	Adult Specific	1.600668	0.794327	0.299845	6.306142	6.302162	6.9949128	56.53006	2.957013	0.441028	6.70482	2.02E-11	1.85E-10	25.50847
Fcer1g	Adult Specific	0.241973	0	0.08571	0.940149	0.421619	0.8591529	8.219521	2.963793	0.988077	2.999557	2.70E-03	0.007343815	136.5015
Plxdc2	Adult Specific	41.84603	44.03818	43.82606	319.7727	304.9748	395.73563	223.0798	2.973474	0.212991	13.96056	2.71E-44	1.68E-42	3.568843
Tnfaip2	Adult Specific	1.076588	1.842956	1.325622	12.00554	11.25524	10.593946	579.9676	2.980448	0.19518	15.27028	1.21E-52	1.12E-50	14.76716
Higd1b	Adult Specific	0.120173	0.0802	0.133022	1.167289	0.523482	0.9067156	15.22617	2.985278	0.664133	4.495002	6.96E-06	3.11E-05	12.7141
Acsm5	Adult Specific	0.436676	0.416319	0.082863	3.084801	1.902187	2.1042109	20.08331	2.988357	0.627534	4.762062	1.92E-06	9.42E-06	831.9986
Kcna7	Adult Specific	0.292706	0.332081	0.304562	2.739774	2.645715	2.0525919	89.2342	2.990879	0.295188	10.1321	3.98E-24	9.63E-23	63.90875
Kcnc3	Adult Specific	0.139183	0.039406	0.005602	0.536306	0.413379	0.3594084	18.41999	3.007686	0.726707	4.13879	3.49E-05	0.000139677	23.25269
Ctla4	Adult Specific	0.050476	0.025666	0.003193	0.178288	0.251287	0.1536181	14.34028	3.01746	0.754318	4.000248	6.33E-05	0.000240956	31.77359
Uts2r	Adult Specific	0.055335	0.098477	0.024501	0.68408	0.482087	0.2947114	4.623477	3.023103	1.17389	2.575286	1.00E-02	NA	63.81415
Slc25a35	Adult Specific	0.659897	1.334525	0.345305	5.82712	7.317037	6.1771169	56.35539	3.024856	0.426726	7.088526	1.36E-12	1.41E-11	22.83925
Lrrc2	Adult Specific	50.42646	39.37999	53.56967	392.5167	389.5505	387.1278	3469.465	3.028762	0.147137	20.58471	3.76E-94	1.08E-91	7.252832
Laptm5	Adult Specific	0.168656	0.107196	0.064008	0.468064	0.892108	1.2832172	19.32526	3.039809	0.626963	4.84847	1.24E-06	6.29E-06	6.254837
P2rx6	Adult Specific	0.447962	0.847039	0.545444	4.944574	4.390534	6.3621721	24.44177	3.050561	0.52925	5.763935	8.22E-09	5.61E-08	10.43576
Crybb1	Adult Specific	0.090347	0.060294	0.060004	0.239338	0.491945	0.9623614	6.579468	3.06202	1.006321	3.042787	2.34E-03	NA	8.112003
Tgtp2	Adult Specific	0.043617	0.135839	0.057936	0.423667	0.85499	0.813052	16.6688	3.062049	0.662775	4.62004	3.84E-06	1.80E-05	1.181498
Iigp1	Adult Specific	5.545865	8.731367	7.274924	60.38565	55.56367	66.18892	2149.675	3.073577	0.170194	18.05927	6.67E-73	1.15E-70	1.226725
Sncg	Adult Specific	0.039283	0.367022	0.052179	1.526267	1.368938	1.2552978	18.31243	3.077809	0.702714	4.37989	1.19E-05	5.14E-05	18.79818
Atp1a2	Adult Specific	48.0513	83.76547	65.92754	590.0905	587.4175	512.90958	12670.82	3.094559	0.178972	17.29077	5.52E-67	7.74E-65	1.108349
Cyp2b10	Adult Specific	0.258104	0.143542	0.05714	1.595404	0.843237	1.1455372	9.779249	3.100658	0.837915	3.700447	2.15E-04	0.000746174	39.73008
Slc1a7	Adult Specific	0.115813	0.020611	0.020511	0.449973	0.554935	0.1644826	8.937264	3.103116	0.931799	3.330241	8.68E-04	0.00264491	3.297902
Slamf9	Adult Specific	0.063484	0.01883	0.018739	0.224233	0.138269	0.4132454	6.466961	3.119683	1.027758	3.035427	2.40E-03	NA	23.19079
Kctd21	Adult Specific	0.205663	0.146403	0.036424	1.089648	1.164647	0.9493027	27.26028	3.137284	0.553305	5.670085	1.43E-08	9.37E-08	7.518506
Mybphl	Adult Specific	0.303037	0.29961	0.313075	2.319132	4.6935	1.1357456	42.01574	3.141189	0.493457	6.365685	1.94E-10	1.60E-09	76.41033
Limch1	Adult Specific	17.5186	31.71013	22.91626	224.37	217.3198	196.81912	5552.628	3.143321	0.18507	16.98448	1.07E-64	1.40E-62	11.79092
Il18bp	Adult Specific	0.24308	0.188085	0.219936	1.735852	1.956611	1.9888502	93.51643	3.144254	0.28908	10.87674	1.49E-27	4.35E-26	19.55476
Igfbp6	Adult Specific	0.10601	0.082539	0.041071	0.280832	0.808127	0.8704304	25.78281	3.148767	0.578322	5.444664	5.19E-08	3.16E-07	67.15226
Pcolce2	Adult Specific	1.702932	2.312837	1.964379	17.33268	16.39785	20.207931	208.6836	3.155232	0.222259	14.1962	9.67E-46	6.65E-44	2.496332
Slco3a1	Adult Specific	4.059455	4.593283	5.003865	43.23696	42.02495	39.01456	859.1244	3.177574	0.153894	20.6478	1.02E-94	2.98E-92	161.6942
Oas2	Adult Specific	7.242583	5.012476	1.068928	31.26666	36.80731	44.288103	24.37679	3.179495	0.58917	5.396563	6.79E-08	4.09E-07	117.8873
Ankrd35	Adult Specific	0.182624	0.073865	0.036755	0.733015	0.687041	1.0021171	25.5277	3.195339	0.571968	5.586568	2.32E-08	1.49E-07	83.33783
Klhl34	Adult Specific	0.008777	0.044908	0.05635	0.426275	0.382334	0.2960583	43.20071	3.215856	0.454938	7.068778	1.56E-12	1.61E-11	27.51027
Smoc2	Adult Specific	0.725183	1.405798	0.860057	8.507668	10.88707	9.1499187	190.0435	3.221579	0.25835	12.46981	1.09E-35	4.67E-34	5.251947
Prss55	Adult Specific	1.014977	0.045157	0	3.943543	2.652779	2.8830384	16.20706	3.239189	1.613009	2.008166	4.46E-02	0.086049676	20.20485
Ephx3	Adult Specific	0.136837	0.03044	0.010098	0.563882	0.447052	0.4048801	10.90324	3.239314	0.857373	3.778187	1.58E-04	0.000560893	2.336662
H2-Q6	Adult Specific	0.044425	0.158122	0.177031	0.863039	1.161114	1.8928466	15.095	3.258086	0.696108	4.680434	2.86E-06	1.38E-05	47.87541
Cngb3	Adult Specific	1.227351	1.110954	0.505954	10.01575	7.835265	8.9411283	109.2937	3.27776	0.316785	10.34695	4.32E-25	1.10E-23	1.281194
Acss1	Adult Specific	13.20391	14.75251	17.72151	147.7261	152.9594	143.25414	4630.038	3.278224	0.143509	22.84328	1.70E-115	9.37E-113	10.81139
Extl1	Adult Specific	3.988533	4.958997	3.725525	41.10624	42.4837	40.157355	389.6989	3.280954	0.185759	17.66243	8.16E-70	1.26E-67	33.19399
Usp18	Adult Specific	0.274293	0.160574	0.230113	1.733718	1.823701	2.7423314	75.29791	3.283726	0.345381	9.507542	1.95E-21	4.05E-20	1.642572
Oas1a	Adult Specific	0.038903	0.060579	0.025837	0.377877	0.29656	0.552516	10.85061	3.285608	0.795669	4.129363	3.64E-05	0.00014496	1.237861
Blk	Adult Specific	0.3706	0.164884	0.302935	1.661437	3.10453	3.137821	47.86294	3.285675	0.4314	7.616303	2.61E-14	3.14E-13	76.15357
Cebpb	Adult Specific	2.64152	5.012476	4.058304	41.3115	33.41078	42.092666	630.3181	3.298801	0.207382	15.90688	5.68E-57	6.01E-55	26.95969
Drd2	Adult Specific	0.16625	0.123278	0.049074	0.831895	1.448394	0.8854388	19.73939	3.3041	0.62165	5.315048	1.07E-07	6.22E-07	3.267175
Ntng2	Adult Specific	0.073636	0	0.048905	0.390137	0.240571	0.457541	5.132764	3.316122	1.183526	2.801899	5.08E-03	NA	92.04544
Rgs7	Adult Specific	0.417474	0.383861	0.135553	2.801697	3.091523	3.1128161	55.75913	3.31863	0.405104	8.192037	2.57E-16	3.71E-15	19.7434
Prlr	Adult Specific	0.537041	0.548906	0.205651	4.178312	4.204562	4.2774788	150.0272	3.321758	0.303716	10.93705	7.67E-28	2.28E-26	1.776897
Chrna10	Adult Specific	0.151836	0.141863	0.04706	1.367581	0.95904	0.9434471	37.11692	3.321937	0.480381	6.915207	4.67E-12	4.57E-11	11.17128
Cyp3a13	Adult Specific	0.737116	0.327951	0.06871	3.425786	3.633412	3.5814604	47.36942	3.357316	0.509267	6.592446	4.33E-11	3.84E-10	13.22887
Ldhd	Adult Specific	0.975432	0.949333	1.498119	12.65094	12.21599	11.47248	203.5939	3.388371	0.237207	14.28844	2.74E-46	1.95E-44	99.39405
P2ry2	Adult Specific	1.410925	2.134304	1.21819	18.31468	16.36393	15.780438	244.3498	3.392295	0.235359	14.41327	4.27E-47	3.16E-45	78.66294
2210407C	Adult Specific	0.076656	0.004872	0.004849	0.2901	0.238513	0.2138529	11.81311	3.40124	0.891573	3.814875	1.36E-04	0.000490713	1.500124
Arc	Adult Specific	0.961405	1.21985	1.009018	12.26267	14.58022	7.3961005	164.0624	3.408684	0.280893	12.13515	6.88E-34	2.77E-32	11.12491
Psmb8	Adult Specific	0.421606	0.364733	0.311123	3.061072	3.724094	4.7819627	84.39127	3.424318	0.323251	10.59337	3.20E-26	8.66E-25	4.273007
Lrg1	Adult Specific	0.546905	1.054404	1.156949	8.907488	11.7794	10.572307	87.37211	3.434473	0.322077	10.66352	1.51E-26	4.17E-25	15.90778
Jam2	Adult Specific	7.931333	9.87769	8.43371	95.29367	97.11906	95.570564	1570.616	3.450551	0.143653	24.02005	1.72E-127	1.29E-124	2.225495
Mme	Adult Specific	1.098074	0.5374	0.63205	10.60137	8.928782	4.3537111	111.5128	3.45102	0.35422	9.742599	1.98E-22	4.36E-21	1.243905
Moap1	Adult Specific	0.106453	0.039468	0.086412	0.752011	0.8115	0.9134351	23.83356	3.488713	0.561047	6.218222	5.03E-10	3.98E-09	26.41832
Ly6c1	Adult Specific	0.463191	0.618237	0.370733	4.971095	6.053068	5.9459247	161.3972	3.539312	0.259345	13.64711	2.10E-42	1.20E-40	23.51953
F13a1	Adult Specific	0.549432	0.36024	0.115234	2.604582	4.156883	4.7743848	67.79475	3.579349	0.429067	8.342162	7.29E-17	1.11E-15	1.499813
Bdh1	Adult Specific	0.701704	0.355556	0.474668	5.748777	5.051984	7.3014318	157.2174	3.620121	0.277763	13.03313	7.93E-39	3.87E-37	89.5554
Fmo2	Adult Specific	0.069302	0.059097	0.012785	0.407976	0.415092	0.8304733	48.50339	3.62463	0.496924	7.294139	3.01E-13	3.30E-12	4.813937
Thrb	Adult Specific	5.115482	4.714437	3.181747	57.21722	58.89169	43.029348	441.2045	3.629385	0.212541	17.07618	2.23E-65	2.96E-63	14.55277
Snai3	Adult Specific	0.121142	0.053898	0.295009	1.283675	2.374665	2.365719	16.73377	3.632862	0.709693	5.118923	3.07E-07	1.70E-06	14.58837
C1qtnf9	Adult Specific	11.14888	22.97385	21.82395	181.3526	233.8798	303.14135	205.9602	3.634568	0.272137	13.35566	1.10E-40	5.90E-39	11.93542
H2-Eb1	Adult Specific	0.102982	0.146617	0.109433	1.30949	1.300929	1.9379473	37.18776	3.639188	0.461925	7.878306	3.32E-15	4.33E-14	117.309
Ccl24	Adult Specific	0.027661	0.006153	0.006124	0.097702	0.180738	0.1473195	5.255443	3.654917	1.180782	3.095337	1.97E-03	NA	12.47805
Lrrc3b	Adult Specific	2.868719	3.509893	3.810532	44.44577	48.84921	40.627191	344.9873	3.69364	0.19668	18.7799	1.10E-78	2.35E-76	182.2897
Hcn2	Adult Specific	1.445146	2.020736	1.645366	21.82416	23.31772	21.990715	382.3187	3.694527	0.1909	19.35322	1.91E-83	4.71E-81	1.494692
Igfals	Adult Specific	0.261563	0.232745	0.221095	3.149583	3.366364	2.7017075	65.19877	3.704919	0.354027	10.46507	1.25E-25	3.30E-24	60.3145
Abcb4	Adult Specific	0.244483	0.257101	0.205018	3.761674	3.130408	2.4924077	425.1802	3.735679	0.205945	18.13919	1.56E-73	2.79E-71	2.002763
Lypd2	Adult Specific	0	0.160656	0	0.956585	0.26216	1.1752771	6.625268	3.738327	1.179727	3.168807	1.53E-03	NA	298.9713

Hspb8	Adult Specific	11.05782	17.27063	18.25125	218.2941	200.1768	215.58203	3669.838	3.760629	0.174555	21.54403	6.02E-103	2.46E-100	11.06769
Slc38a3	Adult Specific	1.875206	1.379376	2.147662	27.46541	25.4313	20.551486	492.217	3.768988	0.204068	18.46928	3.65E-76	7.15E-74	5.88267
Apol9a	Adult Specific	0.119599	0.079816	0	1.267323	0.390736	0.7431399	6.78763	3.773497	1.103477	3.419643	6.27E-04	NA	1.782919
Gbp6	Adult Specific	3.69212	8.635726	6.07194	80.10951	82.02378	97.196253	410.9172	3.77983	0.233787	16.16783	8.50E-59	9.35E-57	33.01104
Klhl33	Adult Specific	0.229338	0.395386	0.355403	4.657814	4.620426	4.7330797	81.62016	3.780534	0.326237	11.58829	4.72E-31	1.65E-29	920.2347
Ifit3	Adult Specific	0.195897	0.271154	0.115649	2.114245	2.891867	3.0140729	61.68787	3.783264	0.39273	9.633234	5.79E-22	1.24E-20	6.529621
Cd52	Adult Specific	0.052134	0	0	0.276219	0.056775	0.1851095	3.414304	3.816458	1.594636	2.39331	1.67E-02	NA	5.784355
Kcnv2	Adult Specific	8.627301	2.709446	0.674099	74.38956	44.21298	45.047474	54.02432	3.83385	0.902115	4.249848	2.14E-05	8.87E-05	81.21156
Scgb1c1	Adult Specific	0.724517	0.671554	0.801983	11.51593	8.679108	11.254626	86.98184	3.839856	0.321753	11.93417	7.85E-33	3.01E-31	9.806936
Xdh	Adult Specific	3.134633	1.722782	1.102168	23.44651	26.10447	33.389689	150.4997	3.884822	0.310584	12.5081	6.74E-36	2.93E-34	36.9271
Grm1	Adult Specific	0.808083	0.742245	0.634795	11.55516	12.20665	8.3762037	205.51	3.894512	0.245556	15.85996	1.20E-56	1.26E-54	6.792419

GSEA Results:

NAME	ES	NES	NOM p-val	FDR q-val	FWER p-val	RANK AT MAX
ADULT_SPECIFIC	-0.42638	-1.40688	0.1031746	0.088272	0.077	2640
NEONATAL_SPECIFIC	0.549873	1.755389	0	0.002466	0	1753

LEADING EDGE

tags=44%, list=18%, signal=54%

tags=25%, list=12%, signal=28%

Suppl. Table 2. KEGG gene sets, downregulated genes, gene set size ≥30

NAME	GS 	GS DETAILS	SIZE	ES	NES	NOM p-val	FDR q-val	FWER p-val	RANK AT M	LEADING EDGE
KEGG_LYSINE_DEGRADATION	KEGG_L	Details ...	41	0.592807	1.683484	0	0.339592	0.2	1585	tags=44%, list=13%, signal=50%
KEGG_FATTY_ACID_METABOLISM	KEGG_F	Details ...	33	0.668033	1.571423	0.012097	0.532321	0.411	1426	tags=61%, list=12%, signal=69%
KEGG_FRUCTOSE_AND_MANNOSE_METABOLISM	KEGG_F	Details ...	30	0.572357	1.551127	0.043564	0.407421	0.438	3025	tags=60%, list=25%, signal=80%
KEGG_PYRUVATE_METABOLISM	KEGG_P	Details ...	31	0.526278	1.524576	0.064202	0.39719	0.547	2345	tags=48%, list=20%, signal=60%
KEGG_PPAR_SIGNALING_PATHWAY	KEGG_P	Details ...	46	0.430718	1.479951	0.045817	0.467289	0.701	1984	tags=43%, list=17%, signal=52%
KEGG_VALINE_LEUCINE_AND_ISOLEUCINE_DEGRADATION	KEGG_V	Details ...	40	0.671066	1.465588	0.032064	0.433986	0.739	1762	tags=68%, list=15%, signal=79%
KEGG_AMINOACYL_TRNA_BIOSYNTHESIS	KEGG_A	Details ...	41	0.534677	1.450052	0.119284	0.414999	0.793	3849	tags=61%, list=32%, signal=90%
KEGG_GLYCEROLIPID_METABOLISM	KEGG_G	Details ...	37	0.430296	1.412065	0.066536	0.471396	0.863	1573	tags=30%, list=13%, signal=34%
KEGG_N_GLYCAN_BIOSYNTHESIS	KEGG_N	Details ...	44	0.455345	1.39876	0.150495	0.450759	0.875	3498	tags=48%, list=29%, signal=67%
KEGG_INSULIN_SIGNALING_PATHWAY	KEGG_I	Details ...	122	0.346128	1.392863	0.032258	0.419585	0.878	2654	tags=34%, list=22%, signal=43%
KEGG_GLYCOLYSIS_GLUCONEOGENESIS	KEGG_G	Details ...	42	0.517369	1.376088	0.163708	0.423962	0.89	2276	tags=55%, list=19%, signal=67%
KEGG_INOSITOL_PHOSPHATE_METABOLISM	KEGG_I	Details ...	50	0.407172	1.307431	0.126984	0.565588	0.959	2671	tags=38%, list=22%, signal=49%
KEGG_ADIPOCYTOKINE_SIGNALING_PATHWAY	KEGG_A	Details ...	58	0.340811	1.290978	0.06917	0.570873	0.976	1531	tags=24%, list=13%, signal=28%
KEGG_GLYCEROPHOSPHOLIPID_METABOLISM	KEGG_G	Details ...	64	0.37544	1.28277	0.210526	0.55534	0.979	2697	tags=44%, list=23%, signal=56%
KEGG_ACUTE_MYELOID_LEUKEMIA	KEGG_A	Details ...	55	0.368043	1.275204	0.117308	0.536679	0.979	3880	tags=49%, list=32%, signal=72%
KEGG_PEROXISOME	KEGG_P	Details ...	70	0.436808	1.268622	0.274354	0.519206	0.981	3125	tags=51%, list=26%, signal=69%
KEGG_NOTCH_SIGNALING_PATHWAY	KEGG_N	Details ...	43	0.389224	1.264049	0.190661	0.501292	0.981	1696	tags=26%, list=14%, signal=30%
KEGG_ARRHYTHMOGENIC_RIGHT_VENTRICULAR_CARDIOMYOPATHY_	KEGG_A	Details ...	57	0.386933	1.256059	0.243564	0.488583	0.985	3171	tags=53%, list=27%, signal=71%
KEGG_AMINO_SUGAR_AND_NUCLEOTIDE_SUGAR_METABOLISM	KEGG_A	Details ...	39	0.378615	1.238459	0.197266	0.504581	0.989	2264	tags=31%, list=19%, signal=38%
KEGG_LYSOSOME	KEGG_L	Details ...	106	0.326542	1.224987	0.231557	0.509073	0.989	1548	tags=25%, list=13%, signal=29%
KEGG_TYPE_II_DIABETES_MELLITUS	KEGG_TYPE_II_DIAI		38	0.360432	1.219934	0.172745	0.496408	0.989	1309	tags=24%, list=11%, signal=27%
KEGG_HYPERTROPHIC_CARDIOMYOPATHY_HCM	KEGG_HYPERTROPI		64	0.336661	1.173646	0.266802	0.572496	0.992	2730	tags=45%, list=23%, signal=58%
KEGG_JAK_STAT_SIGNALING_PATHWAY	KEGG_JAK_STAT_SI		86	0.307079	1.169327	0.190381	0.555459	0.995	1550	tags=21%, list=13%, signal=24%
KEGG_BASAL_CELL_CARCINOMA	KEGG_BASAL_CELL_		36	0.349466	1.146692	0.29783	0.581564	0.998	2389	tags=36%, list=20%, signal=45%
KEGG_CARDIAC_MUSCLE_CONTRACTION	KEGG_CARDIAC_MI		57	0.413135	1.131638	0.341176	0.589924	1	2620	tags=46%, list=22%, signal=58%
KEGG_BASE_EXCISION_REPAIR	KEGG_BASE_EXCISI		32	0.341352	1.120402	0.310345	0.597222	1	3150	tags=38%, list=26%, signal=51%
KEGG_TOLL_LIKE_RECEPTOR_SIGNALING_PATHWAY	KEGG_TOLL_LIKE_R		68	0.275811	1.118124	0.244533	0.580034	1	2072	tags=24%, list=17%, signal=28%
KEGG_PURINE_METABOLISM	KEGG_PURINE_MET		129	0.23339	1.103407	0.28373	0.591158	1	2544	tags=26%, list=21%, signal=33%
KEGG_DILATED_CARDIOMYOPATHY	KEGG_DILATED_CA		69	0.322527	1.097032	0.371951	0.586362	1	2776	tags=43%, list=23%, signal=56%
KEGG_ARGININE_AND_PROLINE_METABOLISM	KEGG_ARGININE_A		42	0.31215	1.088698	0.389113	0.584618	1	1674	tags=33%, list=14%, signal=39%
KEGG_CALCIUM_SIGNALING_PATHWAY	KEGG_CALCIUM_SI		119	0.266505	1.070079	0.38716	0.60083	1	2676	tags=32%, list=22%, signal=41%
KEGG_CYTOSOLIC_DNA_SENSING_PATHWAY	KEGG_CYTOSOLIC_		31	0.278077	1.052943	0.329243	0.616653	1	2738	tags=32%, list=23%, signal=42%
KEGG_PHOSPHATIDYLINOSITOL_SIGNALING_SYSTEM	KEGG_PHOSPHATIL		69	0.307043	1.028095	0.442857	0.646768	1	2822	tags=32%, list=24%, signal=41%
KEGG_RIG_I_LIKE_RECEPTOR_SIGNALING_PATHWAY	KEGG_RIG_I_LIKE_		46	0.254549	0.995019	0.457831	0.695406	1	1032	tags=13%, list=9%, signal=14%
KEGG_ENDOCYTOSIS	KEGG_ENDOCYTOSI		160	0.24393	0.991079	0.45	0.682946	1	2766	tags=26%, list=23%, signal=33%
KEGG_APOPTOSIS	KEGG_APOPTOSIS		73	0.244961	0.978839	0.500994	0.686473	1	1118	tags=14%, list=9%, signal=15%
KEGG_COMPLEMENT_AND_COAGULATION_CASCADES	KEGG_COMPLEMEN		32	0.357117	0.970396	0.506122	0.686111	1	4042	tags=59%, list=34%, signal=89%
KEGG_ALDOSTERONE_REGULATED_SODIUM_REABSORPTION	KEGG_ALDOSTEROI		32	0.294164	0.942225	0.548193	0.720594	1	2590	tags=34%, list=22%, signal=44%
KEGG_NON_SMALL_CELL_LUNG_CANCER	KEGG_NON_SMALL		52	0.266024	0.938276	0.551657	0.710578	1	3967	tags=42%, list=33%, signal=63%
KEGG_MTOR_SIGNALING_PATHWAY	KEGG_MTOR_SIGN/		48	0.274736	0.935289	0.581028	0.697672	1	2505	tags=27%, list=21%, signal=34%
KEGG_PROGESTERONE_MEDIATED_OOCYTE_MATURATION	KEGG_PROGESTERC		73	0.239328	0.93502	0.528689	0.681346	1	3432	tags=34%, list=29%, signal=48%
KEGG_ABC_TRANSPORTERS	KEGG_ABC_TRANSF		30	0.32999	0.926857	0.528487	0.68078	1	2545	tags=50%, list=21%, signal=63%
KEGG_MAPK_SIGNALING_PATHWAY	KEGG_MAPK_SIGN/		209	0.204587	0.884609	0.65286	0.740458	1	2963	tags=28%, list=25%, signal=37%
KEGG_NOD_LIKE_RECEPTOR_SIGNALING_PATHWAY	KEGG_NOD_LIKE_R		44	0.240114	0.803455	0.745902	0.871872	1	3347	tags=36%, list=28%, signal=50%
KEGG_VIBRIO_CHOLERAE_INFECTION	KEGG_VIBRIO_CHO		43	0.193894	0.789756	0.78373	0.875268	1	2776	tags=26%, list=23%, signal=33%
KEGG_LEISHMANIA_INFECTION	KEGG_LEISHMANIA		44	0.211744	0.762049	0.810811	0.896746	1	2703	tags=30%, list=23%, signal=38%
KEGG_DNA_REPLICATION	KEGG_DNA_REPLIC		35	0.215697	0.749998	0.863821	0.894857	1	3112	tags=37%, list=26%, signal=50%
KEGG_WNT_SIGNALING_PATHWAY	KEGG_WNT_SIGNA		122	0.184617	0.73339	0.901575	0.897137	1	2413	tags=22%, list=20%, signal=27%
KEGG_ALZHEIMERS_DISEASE	KEGG_ALZHEIMERS		135	0.195091	0.624804	0.780684	0.988661	1	4076	tags=37%, list=34%, signal=56%
KEGG_ENDOMETRIAL_CANCER	KEGG_ENDOMETRI/		49	0.166942	0.598714	0.913386	0.986378	1	3926	tags=37%, list=33%, signal=54%
KEGG_OXIDATIVE_PHOSPHORYLATION	KEGG_OXIDATIVE_I		96	0.220602	0.514837	0.875	0.999012	1	2878	tags=24%, list=24%, signal=31%
KEGG_PARKINSONS_DISEASE	KEGG_PARKINSONS		102	0.170388	0.42934	0.939024	0.99346	1	2876	tags=23%, list=24%, signal=29%

Suppl. Table 3. Oligos used in this study

Name	Sequence	Purpose/notes
gRNA oligo pool	GCAGATGGCTCTTTGTCCTAG**GCTCTTC**AACCGNNNNNNNNNNNNNNNNNNNNGTTG**GAAGAGC**GTCTTCTGACCGTCGC	14,664 oligos synthesized as a pool. Variable gRNA = NNN. After amplification library was digested with SapI (bold sites), and cloned into CASAAV vector (Addgene 132551).
LibAmp-F	CGTCAGTACAGATCGATTACCTACGGAGTAGACCTGACGCGATGACCTTAACGATAACAGGCAGATGGCTCTTTGTCCTA	Amplification of gRNA oligo pool
LibAmp-R	TTAGCTCGATAGCAGGACAGATCGTTACAGCTTAACTAGCCCAGCCTAGTGTGGATAACGGCGACGGTCAGAAGACGCTC	Amplification of gRNA oligo pool
TnT.titer-F	TCGGGATAAAAGCAGTCTGG	qPCR titer of AAV containing gallus TnT promoter
TnT.titer-R	CCCAAGCTATTGTGTGGCCT	qPCR titer of AAV containing gallus TnT promoter
gRNA.RT	TTTTCAAGTTGATAACGGAC	reverse transcription of gRNAs
gRNA.Adapter-F	AATGATACGGCGACCACCGAGATCTACACTCTTTCCCTACACGACGCTCTTCCGATCTAAGCAGTGGTATCAACGCAG	full length forward adapter, used in first and second round of amplification for gRNA amplicon-seq
gRNA.Adapt.R1-R	CTGGAGTTCAGACGTGTGCTCTTCCGATCTAAGTTGATAACGGACTAGCC	half of reverse adapter, used in first round of amplification for gRNA amplicon-seq
gRNA.Adapt.R2-R	CAAGCAGAAGACGGCATACGAGATNNNNNNGTGACTGGAGTTCAGACGT	second half of reverse adapter, used in second round of amplifcation for gRNA amplicon-seq. NNNNNN = variable multiplexing index.

Suppl. Table 4. Antibodies used in this study.

Antigen	Host	Vendor	Cat. #	Working Concentration	Application
Sarcomeric alpha actinin	Mouse	Abcam	ab9465	1:500	IF
Cav3	Rabbit	Life Technologies	PA1066	1:500	IF
Nppa	Rabbit	Abgent	AP8534A	1:500	IF
H2Bub1	Rabbit	Cell Signaling	5546	1:250; after SDS antigen retrieval	IF
Nppa	Rabbit	Abgent	AP8534A	1:2000	Western
H2Bub1	Rabbit	Cell Signaling	5546	1:1000	Western
GAPDH	Rabbit	Santa Cruz	SC-25778	1:1000	Western
H2Bub1	Rabbit	Cell Signaling	5546	5ul + 90ul Dynabeads	ChIP
H3K4me3	Rabbit	Active Motif	39159	3ul + 90ul Dynabeads	ChIP
H3K36me3	Rabbit	Cell Signaling	4909S	5ul + 90ul Dynabeads	ChIP
