# Supplementary material for: Massively parallel in vivo CRISPR screening identifies RNF20/40 as epigenetic regulators of cardiomyocyte maturation
Source: Nat Commun. 2021 Jul 21;12:4442. doi: 10.1038/s41467-021-24743-z (PMC8295283; doi:10.1038/s41467-021-24743-z)
Supplement: Supplementary file 3 — Reporting Summary [file 41467_2021_24743_MOESM3_ESM.pdf]

nature research

Corresponding author(s): *Double-blind peer review submissions: write DBPR and your manuscript number here instead of author names.*

Last updated by author(s): *YYYY-MM-DD*

Reporting Summary

Nature Research wishes to improve the reproducibility of the work that we publish. This form provides structure for consistency and transparency in reporting. For further information on Nature Research policies, see our Editorial Policies and the Editorial Policy Checklist.

Statistics

For all statistical analyses, confirm that the following items are present in the figure legend, table legend, main text, or Methods section.

n/a	Confirmed	
☐	☒	The exact sample size (*n*) for each experimental group/condition, given as a discrete number and unit of measurement
☐	☒	A statement on whether measurements were taken from distinct samples or whether the same sample was measured repeatedly
☐	☒	The statistical test(s) used AND whether they are one- or two-sided<br>*Only common tests should be described solely by name; describe more complex techniques in the Methods section.*
☐	☒	A description of all covariates tested
☐	☒	A description of any assumptions or corrections, such as tests of normality and adjustment for multiple comparisons
☐	☒	A full description of the statistical parameters including central tendency (e.g. means) or other basic estimates (e.g. regression coefficient) AND variation (e.g. standard deviation) or associated estimates of uncertainty (e.g. confidence intervals)
☐	☒	For null hypothesis testing, the test statistic (e.g. *F*, *t*, *r*) with confidence intervals, effect sizes, degrees of freedom and *P* value noted<br>*Give P values as exact values whenever suitable.*
☒	☐	For Bayesian analysis, information on the choice of priors and Markov chain Monte Carlo settings
☒	☐	For hierarchical and complex designs, identification of the appropriate level for tests and full reporting of outcomes
☐	☒	Estimates of effect sizes (e.g. Cohen's *d*, Pearson's *r*), indicating how they were calculated

Our web collection on statistics for biologists contains articles on many of the points above.

Software and code

Policy information about availability of computer code

Data collection	Guide RNAs were designed using CRISPR RGEN Tools Cas-Database.
Data analysis	Enrichment scores for each gene targeted by the gRNA library were calculated by MAGeCK-0.5.7. No custom code was used.

For manuscripts utilizing custom algorithms or software that are central to the research but not yet described in published literature, software must be made available to editors and reviewers. We strongly encourage code deposition in a community repository (e.g. GitHub). See the Nature Research guidelines for submitting code & software for further information.

Data

Policy information about availability of data

All manuscripts must include a data availability statement. This statement should provide the following information, where applicable:

- Accession codes, unique identifiers, or web links for publicly available datasets
- A list of figures that have associated raw data
- A description of any restrictions on data availability

Data in this publication have been deposited to GEO (GSE139975). The data are also available on the Cardiovascular Development Consortium Server at https://b2b.hci.utah.edu/gnomex/, experiment number 483R (login as guest). The source data underlying bar, violin, and scatter plots in all figures and supplemental figures are provided as a Source Data file.

Field-specific reporting

Please select the one below that is the best fit for your research. If you are not sure, read the appropriate sections before making your selection.

- [x] Life sciences
- [ ] Behavioural & social sciences
- [ ] Ecological, evolutionary & environmental sciences

For a reference copy of the document with all sections, see nature.com/documents/nr-reporting-summary-flat.pdf

Life sciences study design

All studies must disclose on these points even when the disclosure is negative.

Sample size	Group sizes were based on our past maturation studies (Guo and VanDusen et al. 2017. Circ. Research., Guo et al. 2018. Nat. Comms.), in which we show the selected sizes to be sufficient to detect statistically significant differences between groups at moderate and larger biological effect sizes.
Data exclusions	CRISPR screen samples #6, 10, 17, 18, and 30 were excluded for failing to pass our quality control criteria, as described in Supplemental Fig. 2. Excluded samples had poor library coverage, or in the case of sample #18, clustered incorrectly.
Replication	At least three biological replicates were used for each experiment. 11 YFP+ replicates and 14 input control replicates were utilized for the CRISPR screen. The top ten screen hits were individually validated. Seven of the ten robustly validated, while two additional candidates demonstrated weak validation. One candidate was a false positive. We discuss these results in the text.
Randomization	Mice were randomly allocated into experimental groups.
Blinding	Investigators were blinded to group allocation during data collection and analysis.

Reporting for specific materials, systems and methods

We require information from authors about some types of materials, experimental systems and methods used in many studies. Here, indicate whether each material, system or method listed is relevant to your study. If you are not sure if a list item applies to your research, read the appropriate section before selecting a response.

Materials & experimental systems

n/a	Involved in the study	
[ ]	[x]	Antibodies
[x]	[ ]	Eukaryotic cell lines
[x]	[ ]	Palaeontology and archaeology
[x]	[ ]	Animals and other organisms
[x]	[ ]	Human research participants
[x]	[ ]	Clinical data
[x]	[ ]	Dual use research of concern

Methods

n/a	Involved in the study	
[ ]	[x]	ChIP-seq
[ ]	[x]	Flow cytometry
[x]	[ ]	MRI-based neuroimaging

Antibodies

Antibodies used	Sarcomeric alpha actinin (Abcam ab9465), CAV3 (Life Technologies PA1066), NPPA (Abgent AP8534A), H2Bub1 (Cell Signaling 5546), GAPDH (Santa Cruz SC-25778), H3K4me3 (Active Motif 39159), H3K36me3 (Cell Signaling 4909S).
Validation	H2Bub1 - Depleted in Rnf20/40 Cas-KO and RNF20flox KO. ChIP-seq signal marks genes in pattern consistent with previous reports.<br>H3K4me3 - ChIP-seq signal marks active gene TSS's as previously reported.<br>H3K36me3 - ChIP-seq signal marks gene bodies of actively transcribed genes in manner consistent with previous reports.<br>NPPA - Immunostaining and western blot shows increased expression during heart failure, consistent with reports of NPPA expression.<br>CAV3 - Immunostaining shows strong signal at T-tubules, consistent with previous reports.

ChIP-seq

Data deposition

- [x] Confirm that both raw and final processed data have been deposited in a public database such as GEO.
- [ ] Confirm that you have deposited or provided access to graph files (e.g. BED files) for the called peaks.

Data access links
May remain private before publication.

To review GEO accession GSE139975:
Go to https://urldefense.com/v3/__https://www.ncbi.nlm.nih.gov/geo/query/acc.cgi?acc=GSE139975__;!!NZvER7FxgEiBAiR_!7_coAn07inRYtfZKQPa9jFc52VUd4Ksk_ytTY1Le7BJq83at0VXXoehOjXGJ81MkV2t9CskBNSCLbjc$
Enter token ejydikkmjzezdip into the box

Files in database submission

Raw fastq files:
GSM4150667 Screen-S01-input
GSM4150668 Screen-S02-YFP
GSM4150669 Screen-S03-input
GSM4150670 Screen-S04-YFP
GSM4150671 Screen-S05-input
GSM4150672 Screen-S06-YFP
GSM4150673 Screen-S07-input
GSM4150674 Screen-S08-YFP
GSM4150675 Screen-S09-input
GSM4150676 Screen-S10-YFP
GSM4150677 Screen-S11-input
GSM4150678 Screen-S12-YFP
GSM4150679 Screen-S13-input
GSM4150680 Screen-S14-YFP
GSM4150681 Screen-S15-input
GSM4150682 Screen-S16-YFP
GSM4150683 Screen-S17-input
GSM4150684 Screen-S18-YFP
GSM4150685 Screen-S19-input
GSM4150686 Screen-S20-YFP
GSM4150687 Screen-S21-input
GSM4150688 Screen-S22-YFP
GSM4150689 Screen-S23-input
GSM4150690 Screen-S24-YFP
GSM4150691 Screen-S25-input
GSM4150692 Screen-S26-YFP
GSM4150693 Screen-S27-input
GSM4150694 Screen-S28-YFP
GSM4150695 Screen-S29-input
GSM4150696 Screen-S30-YFP
GSM4150697 RNF20/40-Control-S01
GSM4150698 RNF20/40-Control-S02
GSM4150699 RNF20/40-Control-S03
GSM4150700 RNF20/40-Control-S04
GSM4150701 RNF20/40-Control-S05
GSM4150702 RNF20/40-KO-S06
GSM4150703 RNF20/40-KO-S07
GSM4150704 RNF20/40-KO-S08
GSM4150705 RNF20/40-KO-S09
GSM4150706 RNF20/40-KO-S10
GSM4150707 GATA4/6-KO-S01
GSM4150708 GATA4/6-KO-S02
GSM4150709 GATA4/6-KO-S03
GSM4150710 GATA4/6-Control-S04
GSM4150711 GATA4/6-Control-S05
GSM4150712 GATA4/6-Control-S06
GSM4150713 H2Bub1-Input-P1-1
GSM4150714 H2Bub1-ChIP-P1-1
GSM4150715 H2Bub1-Input-P1-2
GSM4150716 H2Bub1-ChIP-P1-2
GSM4150717 H2Bub1-Input-P28-1
GSM4150718 H2Bub1-ChIP-P28-1
GSM4150719 H2Bub1-Input-P28-2
GSM4150720 H2Bub1-ChIP-P28-2
GSM5261295 H3K4me3_P7KO-1_ChIP
GSM5261296 H3K4me3_P7KO-1_Input
GSM5261297 H3K4me3_P7KO-2_ChIP
GSM5261298 H3K4me3_P7KO-2_Input
GSM5261299 H3K4me3_P7Wt-1_ChIP
GSM5261300 H3K4me3_P7Wt-1_Input
GSM5261301 H3K4me3_P7Wt-2_ChIP
GSM5261302 H3K4me3_P7Wt-2_Input

GSM5261303 H3K36me3_P7KO-1_ChIP
GSM5261304 H3K36me3_P7KO-1_Input
GSM5261305 H3K36me3_P7KO-2_ChIP
GSM5261306 H3K36me3_P7KO-2_Input
GSM5261307 H3K36me3_P7Wt-1_ChIP
GSM5261308 H3K36me3_P7Wt-1_Input
GSM5261309 H3K36me3_P7Wt-2_ChIP
GSM5261310 H3K36me3_P7Wt-2_Input

Processed data files:
GSE139975_GATA_RNA-seq.txt
GSE139975_H2Bub1_ChIP-seq.txt
GSE139975_H3K36me3_ChIP-seq.txt
GSE139975_H3K4me3_ChIP-seq.txt
GSE139975_RNF_RNA-seq.txt
GSE139975_screen_gRNA.txt
GSE139975_screen_gene.txt

Bigwig files for all ChIP-seq samples:
GSE139975_RAW.tar

Genome browser session (e.g. UCSC)

Provide a link to an anonymized genome browser session for "Initial submission" and "Revised version" documents only, to enable peer review. Write "no longer applicable" for "Final submission" documents.

Methodology

Replicates

14 input control samples and 11 YFP(+) CRISPR screen samples passed our quality control criteria, as described in SuppFig-02. These samples had appropriate gRNA distribution and clustered into separate input and YFP(+) groups (Fig-01). Two biological replicates were used for each group in each ChIP-seq experiment. Replicate correlations were high for all groups (Fig-06, SuppFig-07). Three biological replicates were used for each group in the P0/P28 cardiomyocyte RNA-seq experiment (SuppFig-06), while five biological replicates were used for each group in the Cas-RNF20/40 KO RNA-seq experiment (Fig-04). PCA analyses showed distinct and consistent molecular signatures for each group.

Sequencing depth

Single end sequencing was used for all experiments. The 25 CRISPR screen samples had an average sequencing depth of 4.8M reads. ChIP-seq and RNA-seq samples had >20M reads.

Antibodies

Describe the antibodies used for the ChIP-seq experiments; as applicable, provide supplier name, catalog number, clone name, and lot number.

Peak calling parameters

N/A

Data quality

ChIP-signal was quantified at each gene body as described in the methods (no peaks were called). As correlations between replicates were high (see above), the average RPKM normalized signal at each gene was used in analyses.

Software

Adapters were trimmed from the 5' end of reads, and the 3' end was trimmed by quality score (bases scoring < 28 removed) using Trimmomatic/v0.36. Trimmed reads were aligned to mm10 using Bowtie2/v2.3.4.3. Indexed Bam files were generated using Samtools/v1.9. Bigwig files were generated using Deeptools/v3.0.2. Reads at gene bodies were quantified using Bedtools/v2.27.1.

Flow Cytometry

Plots

Confirm that:

- [x] The axis labels state the marker and fluorochrome used (e.g. CD4-FITC).
- [x] The axis scales are clearly visible. Include numbers along axes only for bottom left plot of group (a 'group' is an analysis of identical markers).
- [x] All plots are contour plots with outliers or pseudocolor plots.
- [x] A numerical value for number of cells or percentage (with statistics) is provided.

Methodology

Sample preparation

CMs were dissociated by retrograde collagenase perfusion. CMs were enriched by centrifugation at 20g for 5 minutes, and then resuspended in PBS for sorting.

Instrument

Sorting was performed at the DANA Farber Cancer Center flow cytometry core on an Aria II cell sorter with 100 µm nozzle, 510/21 bandpass filter for GFP, 550/30 bandpass filter for YFP, and 525 longpass dichroic filter to split GFP and YFP signals.

Software

.fcs files were analyzed with FlowJo.

Cell population abundance	In screen samples YFP(+) cells typically made up ~10% of the total number of GFP(+) CMs, which composed ~ 50% of the total number of CMs. Post-sort purity was confirmed by analyzing the sorted population by FACS (>98% purity), and by RNA-sequencing in the Cas-RNF20/40 KO experiment (~3.8 fold overexpression of Myh7 in Myh7YFP(+) CMs versus GFP(+) CMs.
Gating strategy	Cardiomyocytes are large cells easily identified by gating events with high SSC-A and FSC-A. In addition, both markers that we sort from, GFP and YFP, are cardiomyocyte specific. From the high SSC-A/FSC-A population GFP(+) CM could clearly be distinguished (Fig03-F, leftmost panel). The GFP(+) CMs could further be sorted into YFP(+) and YFP(-) by setting the gate boundary with a Cre-transduced negative control sample (Fig03-F, right panels).

☒ Tick this box to confirm that a figure exemplifying the gating strategy is provided in the Supplementary Information.
