# Supplementary material for: Massively parallel in vivo CRISPR screening identifies RNF20/40 as epigenetic regulators of cardiomyocyte maturation
Source: Nat Commun. 2021 Jul 21;12:4442. doi: 10.1038/s41467-021-24743-z (PMC8295283; doi:10.1038/s41467-021-24743-z)
Supplement: Supplementary file 4 — Description of Additional Supplementary Files [file 41467_2021_24743_MOESM4_ESM.pdf]

Description of Additional Supplementary Files

Supplementary Data 1: GATA4 regulated genes in neonatal heart. GATA4/6 RNA-seq: A high dose of AAV-Cre was given to P0 Gata4fx/fx;Gata6fx/fx pups. CMs were isolated and reporter positive cells FACS sorted at P6. Tab1) There were 347 genes differentially expressed with p-adj < 0.05 and base mean expression in the control group > 5 (164 downregulated, 183 upregulated). 30 of the 347 were excluded due to their not being annotated in CRISPR RGEN gRNA design tool. 58 of the 247 were excluded due to their already being present in the transcriptional regulator candidate list. Tab 2) 1554 gRNAs targeting the remaining 259 genes were included in the library GATA4 ChIP-seq: GATA4-flagbio ChIP-seq was conducted at P0 on cardiac apex tissue. Peaks were assigned to the nearest gene, and given a score based on GATA4 binding strength. Tab 3) Genes associated with the top 471 peaks were selected. 97 of the 471 genes were excluded due to their not being annotated in CRISPR RGEN gRNA design tool. 29 of the 471 genes were excluded due to their already being present in the GATA4/6 CKO RNA-seq list. 54 of the 471 genes were excluded due to their already being present in the transcriptional regulator candidate list. Tab 4) 1746 gRNAs targeting the remaining 291 genes were included in the library.

Supplementary Data 2: gRNA sequences. 14675 guide RNAs were synthesized as an oligonucleotide pool. The oligonucleotidues were 80 nt long and corresponded to the following template, with _N20_ being replaced by the individual 20 nt guide sequences listed in the table. Class indicates the reason that the gene was included in the library: TR, transcriptional regulator; GATA Target, Gata targets; NC, human negative control.

Supplementary Data 3: Final genetic screen results. Final results of the screen are summarized at the level of individual gRNAs ("FINAL_25samp_DESeq2") and at the level of individual genes ("FINAL_25samp_MaGeCK"). "Group" indicates whether the gRNA originated from Transcriptional Regulators (TR) or GATA4 targets. On The MaGeCK tab, the number of significantly enriched or depleted individual gRNAs targeting that gene is shown under the "num_enriched" and "num_depleted" columns.

Supplementary Data 4: RNA-seq of mosaic CASAAV depletion of RNF20/40. DESeq2 analysis of RNA-seq on RNF20/40 mosaic depletion in cardiomyocytes by CASAAV. Myh7YFP/+; R26fsCas9-P2A-GFP mice were treated with CASAAV-RNF20/40 or AAV-Cre without gRNAs at P1. The virus was dosed so that approximately 20% of cardiomyocytes would be transduced. GFP+ (Cre) or YFP+ (CASAAV-RNF20/40) CMs were purified by FACS. RNA isolated from cells was used for RNA-seq (n=5 per group). Data were anayzed using DESeq2.

Supplementary Data 5. Differential gene expression between neonatal and mature CMs. Cardiomyocytes were purified from P1 or P28 hearts. Transcripts were quantified by RNA-seq. Differential gene expression was analyzed by DESeq2. To define the top or bottom 100 ranked genes, genes were ranked by Log2FC(P28/P1). Genes with maximum FPKM across all samples <= 1 were excluded.

Supplementary Data 6. H2Bub1 ChIP-seq signal on gene bodies. H2Bub1 ChIP-seq and input reads mapping within gene bodies were counted and normalized using DESeq2. Normalized counts as reads per million were divided by gene body length in kb.
